# Genome-wide transcriptional effects of deletions of sulphur metabolism genes in *Drosophila melanogaster*

**DOI:** 10.1016/j.redox.2020.101654

**Published:** 2020-07-25

**Authors:** O. Zatsepina, D. Karpov, L. Chuvakova, A. Rezvykh, S. Funikov, S. Sorokina, A. Zakluta, D. Garbuz, V. Shilova, M. Evgen'ev

**Affiliations:** a Engelhardt Institute of Molecular Biology of Russian Academy of Sciences, Moscow, Russia; b Moscow Institute of Physics and Technology, Dolgoprudny, Moscow Region, Russia; c Koltzov Institute of Developmental Biology of Russian Academy of Sciences, Moscow, Russia

**Keywords:** H_2_S-Producing genes, CRISPR/Cas9, Deletions, *Drosophila melanogaster*, RNA-Seq

## Abstract

In recent years, the gasotransmitter hydrogen sulphide (H_2_S), produced by the transsulphuration pathway, has been recognized as a biological mediator playing an important role under normal conditions and in various pathologies in both eukaryotes and prokaryotes. The transsulphuration pathway (TSP) includes the conversion of homocysteine to cysteine following the breakdown of methionine. In *Drosophila melanogaster* and other eukaryotes, H_2_S is produced by cystathionine β-synthase (CBS), cystathionine γ-lyase (CSE), and 3-mercaptopyruvate sulphurtransferase (MST). In the experiments performed in this study, we were able to explore the CRISPR/Cas9 technique to obtain single and double deletions in homozygotes of these three major genes responsible for H_2_S production in *Drosophila melanogaster*. In most cases, the deletion of one studied gene does not result in the compensatory induction of two other genes responsible for H_2_S production. Transcriptomic studies demonstrated that the deletions of the above *CBS* and *CSE* genes alter genome expression to different degrees, with a more pronounced effect being exerted by deletion of the *CBS* gene. Furthermore, the double deletion of both *CBS* and *CSE* resulted in a cumulative effect on transcription in the resulting strains. Overall, we found that the obtained deletions affect numerous genes involved in various biological pathways. Specifically, genes involved in the oxidative reduction process, stress-response genes, housekeeping genes, and genes participating in olfactory and reproduction are among the most strongly affected. Furthermore, characteristic differences in the response to the deletions of the studied genes are apparently organ-specific and have clear-cut sex-specific characteristics. Single and double deletions of the three genes responsible for the production of H_2_S helped to elucidate new aspects of the biological significance of this vital physiological mediator.

## Graphical abstract

**Figure:**
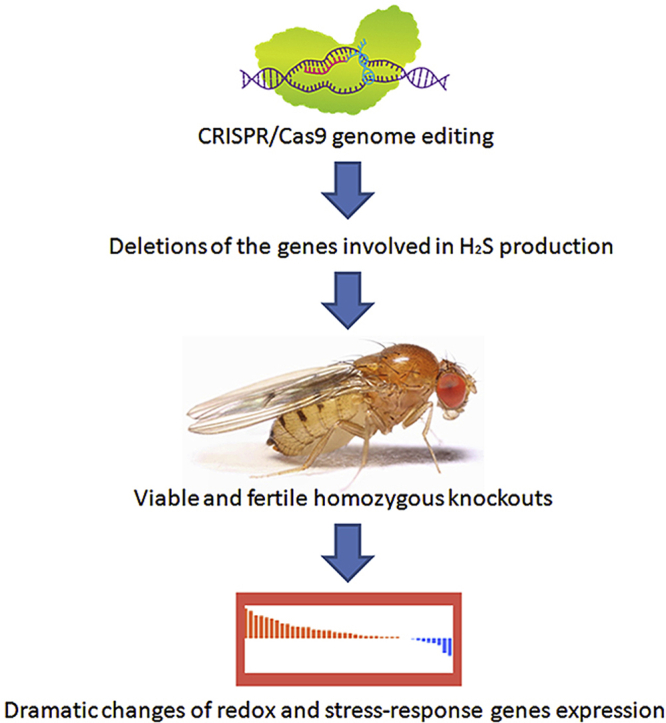

## 1 Introduction

In recent years, the gasotransmitter hydrogen sulphide (H_2_S) has been recognized as a biological mediator of immense importance both in eukaryotes and prokaryotes [[1], [2], [3]]. H_2_S is produced in cells mostly through the reverse transsulphuration pathway (TSP). Transsulphuration is a vital metabolic process common to prokaryotes and eukaryotes studied in detail in mammals and several other organisms. TSP includes the conversion of homocysteine to cysteine following the breakdown of methionine [[2], [3], [4], [5]]. H_2_S plays various roles in several vital processes, including neuromodulation, cytoprotection, anti-inflammation, angiogenesis and regulation of vascular tone [6,7]. In eukaryotes, H_2_S is produced by cystathionine β-synthase (CBS), cystathionine γ-lyase (CSE), and 3-mercaptopyruvate sulphurtransferase (MST). In essence, MST, which is localized in both the cytoplasm and mitochondria, acts as a sulphur carrier, rather than an H_2_S producer [6].

CBS is the first rate-limiting enzyme in TSP and produces H_2_S and the cysteine precursor cystathionine by utilizing homocysteine [2,8]. Non-protein sulphur amino acid homocysteine is converted to cystathionine via cystathionine-β-synthase (CBS) or is remethylated using methionine synthase [9]. In cancer cells, silencing of the *CBS* gene severely reduces cellular glutathione (GSH)) levels, impairs H_2_S production, activates tumour suppressors, such as p53, and inhibits NF-kB activation [8]. CSE is another enzyme that plays an important role in cysteine metabolism and H_2_S production. Homocysteine serves as a substrate for CSE, leading to the production of H_2_S, a-ketobutyrate, ammonia, homolanthionine and cystathionine, which serves as a CSE substrate for cysteine production [10]. These metabolic pathways in mammals are expressed in different tissues during ontogenesis, and the human brain, liver and muscle tissues are the primary sites of activity for these enzymes [2,11]. Model animals deficient in *CBS* developed hyperhomocysteinemia and represent an excellent tool to monitor the effects of genes involved in methionine metabolism during pregnancy and the development of various pathologies [11]. Furthermore, the effect of H_2_S on the hallmarks of ageing and several age-related pathologies has been revealed in various organisms and model species [12]. It is also known that H_2_S production plays a critical role in yeast, *Drosophila* and nematode models of dietary restriction (DR)-mediated longevity [13,14]. It was demonstrated by several groups that DR can upregulate hydrogen sulphide and block mitochondrial oxidative stress [15,16]. While the induction of H_2_S synthesizing enzymes appears to be a conserved and essential feature of the caloric restriction (CR) response in evolutionarily distant organisms, such as worms, flies and mice [2,14,15,17], the mechanism of the signalling pathway-mediated cytoprotective functions of H_2_S is not well understood. Modulation of the TSP was found to determine the impact of diet on overall protein translation and homeostasis [4,14,15]. It was shown that patients with genetic defects in the TSP are characterized by high levels of homocysteine, low levels of GSH and increased incidence of age-related pathologies [4,12]. There is growing evidence in favour of cross-talk between H_2_S and NO levels playing an important role in cardioprotection [2,18], as well as the interaction between TSP and another ancient stress-response system comprising heat shock proteins (Hsps) induced by many stimuli in various organisms [19].

It was demonstrated by different groups that defects of the H_2_S synthesizing enzyme system are involved in a plethora of diseases in humans, including cancer and different neurodegenerative diseases [6,8].

There are several reports describing the effect of CBS or CSE knockdown or overexpression on various physiological parameters. Thus, it was shown that overexpression of CSE in a *Drosophila* model suppresses several detrimental effects of spinocerebellar ataxia type 3 (SCA3). Specifically, CSE overexpression in this model apparently ameliorated the downstream consequences of protein aggregation, preventing SCA3-induced tissue degeneration [10]. Furthermore, the reduction of CBS levels induces premature senescence in human endothelial cells [20]. It was also shown that strong, constitutive expression of *CBS* RNAi in *D. melanogaster* resulted in death during development [4]. Therefore, in these experiments, the researchers targeted RNA-mediated knockdown of *CBS* to the adult stage by using the gene-switch inducible expression system [22,21]. In diet-restricted flies, these authors observed an increase in CBS protein and corresponding mRNA, which indicates significant transcription upregulation [4].

Cysteine synthesis is the rate-limiting step in the production of glutathione (GSH), which is the ubiquitous antioxidant found in various organisms [2]. Notably, GSH is a downstream metabolite of TSP, and its synthesis is dependent on the availability of cysteine. GSH levels were increased in the diet-restricted flies. After the work of Kabil et al. [4], it was widely accepted that knockdown of *CBS* is lethal in fruit flies [9]. However, the lethality observed in the case of *CBS* RNAi-mediated knockdown in *D. melanogaster* [4] is probably due to another second site or sites of lethal mutations induced as a by-product of RNAi experiments performed by this group. In our studies exploring the CRISPR/Cas9-based approach, we were able to develop *D. melanogaster* containing homozygous deletions of the *CBS*, *CSE* and *MST* genes. Moreover, we obtained flies comprising homozygous deletions of both *CBS* and *CSE* genes (i.e., double deletions). Importantly, all these lines with deletions were viable and fertile, although they exhibited certain defects in development and lifespan parameters (paper in preparation).

In this study, we report the results of analysis of the transcriptomic changes that occurred in *D. melanogaster* strains containing the deletions of the three major genes (*CBS*, *CSE* and *MST*) involved in methionine metabolism and H_2_S production. The accumulated data strongly suggest that single and double deletions of the above *CBS* and *CSE* genes, particularly *CBS*, dramatically alter the normal function of several diverse systems and result in transcriptome remodelling of *D. melanogaster*.

## 2 Materials and methods

**Drosophila stocks and maintenance**. In our study, to develop flies with deletions we used stock 58492 with genotype y1 M{Act5C-Cas9.P.RFP-}ZH-2A w1118 DNAlig4169 obtained from the Bloomington *Drosophila* Stock Center and transgenic *CBS−/−*, *CSE−/−*, *MST−/−* and (*CBS−/−*, *CSE−/−*) strains developed in our laboratory. Flies (separately males and females) were maintained at 23 °C on standard yeast, sugar, and agar medium in 40 ml vials at a density of ~20 flies per vial throughout the experiment. All flies were synchronized by age: enclosing individuals were collected daily, transferred to new vials with medium, and then aged appropriately.

**The deletion of CBS gene in flies using CRISPR/Cas9 system**. Construction of the system for *CBS* gene deletion by the integration of the mCherry expressing reporter construct has been previously described in detail [19]. To make sure there are no off-target sites of integrated reporter construct in transgenic flies, Southern blot analysis was performed. Isolation of genomic DNA from adult flies and Southern blot analysis to detect new insertions in *CBS* gene was performed according to Evgen'ev et al. [23]; Shilova et al. [19].

Five micrograms of each DNA sample was digested with *Bam*HI/*Pst*I restriction endonucleases. *mCherry* gene was labeled by random priming and used as probe for standard high-stringency hybridization (Suppl. Fig 1A). Southern blotting did not reveal any off-target sites with integrated reporter construct in the obtained three strains with deleted *CBS* gene. In the following experiments we used two strains (*CBS−/−*5 and *CBS−/−*8) that did not comprise off-target sites.

**Fig. 1:**
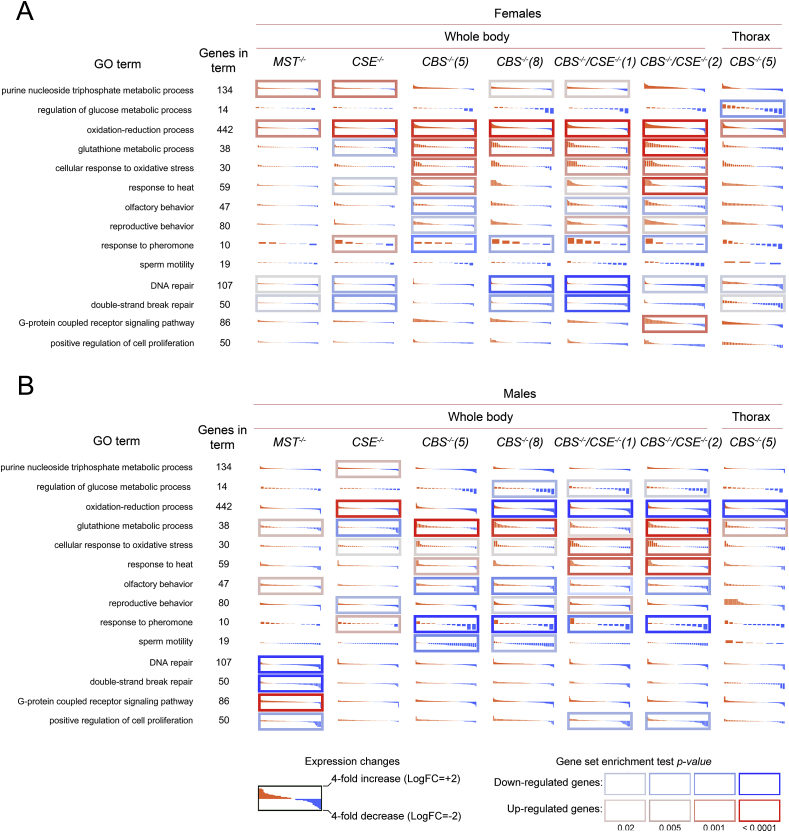
Gene ontology (GO) and pathway analysis of differentially expressed genes in females - A, males - B. Expression level changes induced by single *CBS, CSE, MST* and double deletions (*CBS−/−*, *CSE−/−*) in females and males. Each cell represents the sorted binary logarithms of expression value fold changes (LogFC) in the mutant lines versus control species for genes participating in a specific GO pathway. LogFC (vertical axis) ranges from -2 to ±2, i.e., -2. from a 4-fold decrease (blue) to a 4-fold increase (red). Cell borders demonstrate the statistical significance of gene set enrichment analysis (Fisher test *p*-value): blue (enriched with downregulated genes) and red (enriched with overexpressed ones). (For interpretation of the references to colour in this figure legend, the reader is referred to the Web version of this article.)

**Construction of the system for CSE gene deletion**. For *CSE* gene deletion two plasmids were generated: pAc-CSE-dual-sgRNA plasmid carrying dual spacers targeting 5′- and 3′-regions of the *CSE* gene and pSK-mCherry-CSE integration plasmid. For generation pAc-CSE-dual-sgRNA plasmid *CSE* gene sequence (CG5345) was obtained using Flybase [24]. Target regions in *CSE* gene were chosen as having low nucleosome occupancy according to data obtained in Ref. [25] and visualized in the UCSC browser (https://genome.ucsc.edu/) [26]. Sequences of the target regions were amplified with the pairs of primers CSE_CG5345-cyto-5′-flank-genome-check-F/CSE_CG5345-cyto-5'_ex1-white-4_in-R and CSE_CG5345_cyto-3′-flank-*Not*I-F/CSE_CG5345_cyto-3′-flank-genome-check-R followed by Sanger sequencing. Resulting sequences were used to design spacers for the CRISPR/Cas9 system using CRISPOR (http://crispor.tefor.net/) [27]. High-ranked spacers having the least possible off-targets were chosen for further cloning into pAc-dual-sgRNA plasmid described in Ref. [28]. Fragment including full first sgRNA, U6-1 terminator, U6-2 promoter, and spacer for the second sgRNA was amplified with the pair of primers CSE_CG5345_cyto-5′-flank-sgRNA-F/CSE_CG5345_cyto-3′-flank-sgRNA-R using pAc-dual-sgRNA as a template. PCR product was cloned into *Bbs*I-cut pAc-dual-sgRNA using Gibbson assembly [29]. The correctness of the dual-sgRNA construct was verified by Sanger sequencing. Integration plasmid for *CSE* gene deletion carrying mCherry as a reporter (pSK-mCherry-CSE) was constructed as follows. Up-flank of *CSE* gene fused to 5′-region of 4th intron from *white* gene was amplified by overlap PCR using primers CSE_CG5345-cyto-5′-flank-*Xba*I-F, CSE_CG5345-cyto-5'_ex1-white-4_in-F, CSE_CG5345-cyto-5'_ex1-white-4_in-R, CSE_CG5345-cyto-5′-white-4_in-*Xho*I-R and cloned into pSK-mCherry integration vector described in Ref. [27] at *Xba*I/*Xho*I sites. Down-flank of *CSE* gene was amplified with primers CSE_CG5345-3′-*Eag*I-flank-F/CSE_CG5345-3′-*Sac*I-flank-R and cloned into pSK-mCherry integration vector at *Eag*I/*Sac*I sites. The correctness of inserts was verified by Sanger sequencing.

**Construction of the system for MST gene deletion**. For *MST* gene deletion, two plasmids were generated: pAc-MST-sgRNA plasmid carrying spacer against *MST* and pSK-mCherry-MST integration plasmid. Plasmids were obtained using the experimental pipeline as described for the *CSE* gene. Target regions of *MST* (CG12279) were amplified and Sanger sequenced using pairs of primers: MST_CG12279-5′-flank-*Xba*I/MST_CG12279-5′-flank-*Xho*I-R and MST_CG12279-3′-flank-*Eag*I-F/MST_CG12279-3′-flank-genome-check-R. pAc-MST-sgRNA plasmid was obtained by annealing pair of the oligonucleotides sgRNA-MST-5′-ol-F/sgRNA-MST-5′-ol-R and cloning them into *Bbs*I-cut pAc-dual-sgRNA. Integration plasmid for the *MST* gene deletion was obtained by cloning up-flank amplified with primers MST_CG12279-5′-flank-*Xba*I/MST_CG12279-5′-flank-*Xho*I-R and down-flank amplified with primers MST_CG12279-3′-flank-*Eag*I-F/MST_CG12279-3′-flank-*Sac*I-R. Primers used in CRISPR/Cas9-based experiments are given in Table S1.

**Embryo injection**. Preblastoderm embryos of *Drosophila melanogaster* strain 58492 with genotype given above were used for injection as described in Zhang et al. [30]. We used a 500 ng/μl mixture of two plasmids, sgRNA coding plasmid: pAc-CSE-dual-sgRNA or pAc-MST-sgRNA and homologous pSK-mCherry-*CSE* or pSK-mCherry-*MST* integration plasmid (1:5). A total of 300 embryos were injected. Two-hundred adults that developed from injected embryos were out crossed to laboratory strain *yw* (df (1)w, yw67c23 and flies carrying the gene deletion were selected based on the expression of the *mCherry* gene under control of the actin 5C promoter. Two strains contained *CSE* deletion and nine strains containing *MST* deletion were obtained in our injection experiments.

**Verification of constructs integration**. Integration of the mCherry containing integration constructs into *CSE* or *MST* locus was checked by PCR using primers for 5′-site of the *CSE* gene - CSE_CG5345-cyto-5′-flank-genome-check-F and 5′-flank-check-R or for 5′-site of the *MST* gene - MST_CG12279-5′-flank-genome-check-F and 5′-flank-check-R; for 3′-site of the *CSE* gene for 3′-site of the *CSE* gene - CSE_CG5345_cyto-3′-flank-genome-check-R and 3′-flank-check-F or for 3′-site of the *MST* gene - MST_CG12279-3′-flank-genome-check-R and 3′-flank-check-F (Table S1) using genomic DNA To make sure that there are no off-target sites with the integrated reporter construct in the transgenic strains containing *CSE* and *MST* deletions, Southern blot analysis with genomic DNA of these strains was performed. The DNA from flies with deleted *CSE* gene was digested with *Bam*HI restriction endonuclease, with deleted *MST* gene was digested with *Eco*RI restriction endonuclease. *mCherry* gene was labeled by random priming (^32^P) and used as probe for standard high-stringency hybridization (Sup. Fig. 1A and B). *CSE−/−*(1) transformant (line5) does not contain off-targets but *CSE−/−*(2) transformant (line 6) carries a single off-target insertion and, hence, we used *CSE−/−*(1) strain for the analysis. From nine transformants with deleted *MST* gene four did not carry off-targets and one of them was selected for further studies. To verify the deletion of *CSE* or *MST* genes qRT-PCR studies were performed using DNA and RNA from the strains with deletions using specific primers (Table S2).

**The development of flies with double deletions (CBS and CSE)**. To obtain double deletions, 2 independent *CBS* transformant strains – *CBS−/−*(5) and *CBS−/−*(8) and one *CSE−/−*(1) transformant (without off-targets) were used. We also used two strains carrying the balancers yw; CyO/If – X chromosome and Df(1)260-1, y [1]/FM4 – second chromosome. As a result of five subsequent crosses, double homozygotes were obtained: *CBS−/−*, *CSE−/−*(1) from crosses of *CBS−/−*(5) with *CSE−/−*(1), and *CBS−/−*, *CSE−/−*(2) from crosses of *CBS−/−*(8) and *CSE−/−*(1). The scheme of crosses used to develop flies comprising double deletions is present in Supplementary Materials. The double deletion strains were checked by Southern blot hybridization with probes for *CBS* and *CSE* genes (Figure S1A, B).

**Measurements of Cellular Glutathione Levels**. Cellular GSH concentrations were determined using Ellmann's reagent as described elsewhere [[31], [32],^33^]. Briefly 5d males and females were frozen in liquid nitrogen and homogenized in 20 mM Tris-HCl pH 8 at the presence of protease inhibitor cocktail. Protein concentration was determined using Bradford protein assay method [34] and proteins were precipitated by 50% TCA. Nonprotein thiol levels (GSH being a major component) in the supernatant were determined by adding Ellmann's reagent (5,5-dithiobis(2-nitrobenzoic acid)) and assessing optical density (412 nm; Jasco v-560 spectrophotometer, PerkinElmer). GSH content was calculated basing on the measured protein concentration in the individual probes. Each point represents an average of at least five independent biological replicates.

**RNA extraction and quantitative real-time PCR**. Procedures were identical to those described in Shilova et al. [35]. Briefly: total RNA was extracted from whole adult flies of all investigated straines or thoraxes from *CBS−/−*(5) and control straines using guanidine isothiocyanate RNAzol RT (Molecular Research Center, USA) following the manufacture's protocol. One microgram of total RNA was used for cDNA synthesis with an MMLV RT kit (Evrogen, Russia). All qRT-PCR reactions were conducted using the SYBR Green fluorescent dye (Evrogen, Russia) in an ABI PRISM VR 7500 device (Applied Biosystems, USA). The relative expression of studied genes was calculated based on the ΔΔ*C*t method [36]. Quantifications were normalized to the housekeeping gene rp49 [37]. All experiments were performed with three to five biological replicates and three experimental replicates. The primers used in qRT-PCR experiments are given in Table S2.

**RNA-seq libraries preparation and data analysis**. Total RNA extraction from whole adult flies and thoraxes were performed as described in the previous section. The concentration of RNA was measured with a Qubit Fluorometer (Invitrogen, USA). The quality of RNA was determined with an Agilent BioAnalyzer 2100 using an RNA 6000 nano kit. The RNA Integrity Number (RIN) of all RNA samples taken for mRNA libraries preparation was not less than 8. Libraries for RNA-seq were prepared using the NEBNext Ultra II Directional RNA Library Prep Kit for Illumina (New England Biolabs, USA) according to the manufacturer's guidelines. Seventy-five bp single-end sequencing was conducted on an Illumina NextSeq 500 platform.

As a result of deep-sequencing, we obtained ~15 million reads for each library. Processing of raw sequence data was performed using PPLine script (PMID: 26147802) included mapping of reads to the *D. melanogaster* genome (release dm6) with STAR (PMID: 23104886) after adapter, length and quality trimming by Trimmomatic (PMID: 24695404). Differential gene expression analysis was performed with the edgeR package (PMID: 19910308). Gene Ontology and KEGG enrichment analyses were performed using topGO (v.2.36.0) and clusterProfiler Bioconductor packages (PMID: 22455463). Visualization of gene set enrichment analysis (GSEA) was performed using custom scripts written in Python and R.

Sequence data were deposited in NCBI GEO database under the number GSE148109.

## 3 Results

**Complex interaction between major genes responsible for H_2_S production and GSH metabolism**. To determine whether there is some feedback interaction between CBS, CSE and MST expression, we compared the levels of corresponding RNAs in the CRISPR/Cas9-generated strains comprising single and double deletions of these genes by exploring Illumina-based transcriptomic data and qRT-PCR approaches. The level of *CBS* transcription was not changed in the strains containing *CSE* or *MST* deletions (Figure S3). Interestingly, the level of *CSE,* and especially *CBS* expression, was several times higher in the females of all studied strains (Figure S4). We failed to detect any significant compensatory increase in *CBS* or *MST* expression in the strains with knockdown of the *CSE* gene. Similarly, we failed to reveal any modulation of *MST* expression in the strains with deletions of *CBS* or *CSE* (Figure S4). On the other hand, in the case of the strain with *CBS* deletion, while we failed to observe any compensatory effect in terms of *CSE* or *MST* expression in the females of this strain, we revealed a two-fold compensatory increase in *CSE* expression in the males of *CBS−/−* strains (Figure S3, S4).

To determine what cellular processes are affected in the strains containing single and double deletions of the above three genes involved in H_2_S production and methionine metabolism, transcriptomic analysis exploring the control strain and flies of both sexes containing single and double deletions of *CBS*, *CSE* and *MST* was carried out. Specifically, the deletion of *CBS* affected the expression of the genome significantly more strongly in comparison with *CSE−/−* and *MST−/−* deletions. Strains with double deletions (*CBS−/−*, *CSE−/−*) largely have rather similar patterns with *CBS−/−* strains, but the observed changes are more dramatic, suggesting a cumulative effect of these two gene deletions (Fig. 1).

**Fig. 2:**
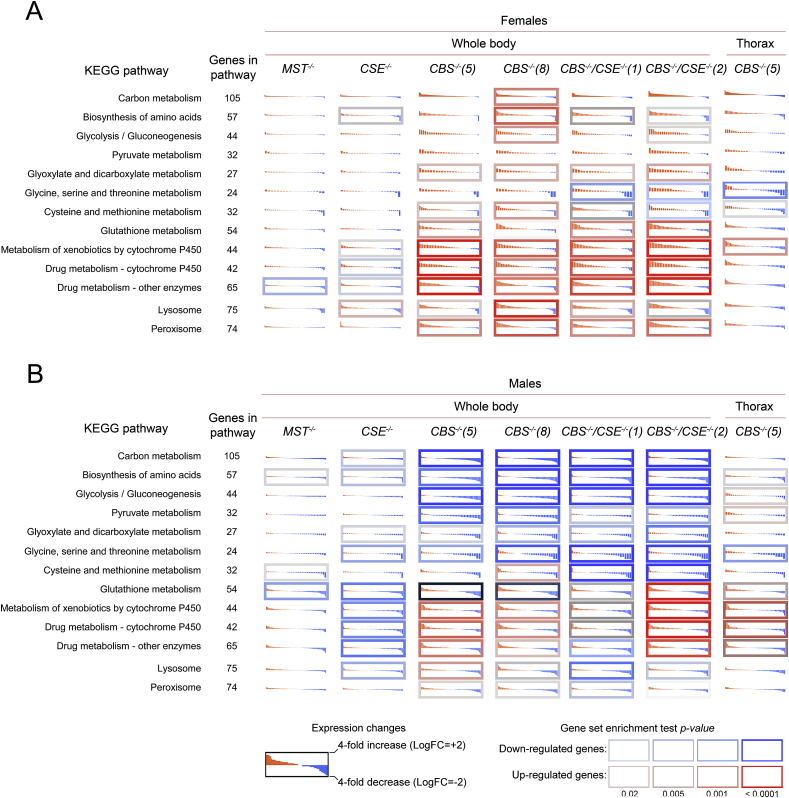
KEGG pathways. Differential expression profiles of genes participating in the most affected KEGG pathway deletions. The effects of single deletions of *CBS, CSE, MST* and double deletion (*CBS−/−*, *CSE−/−*) genes in whole body females/males and thoraxes for *CBS−/−(5)* are depicted. Red and blue subplots illustrate expression level changes (log-scale; values are sorted in each subplot) between mutant and control lines. (A) females; (B) males. The log expression level fold change (LogFC) range is from -2 (i.e., four-fold downregulation; blue) to ±2 (i.e., fourfold overexpression; red). Cell borders indicate the gene set enrichment (Fisher's exact test) *p* value for a pathway. The red border indicates that a KEGG pathway is enriched with upregulated genes; the blue border indicates downregulated genes. *min.p* (*up/downreg*) – minimal gene set enrichment test (Fisher's exact test) *p* value for up/downregulated genes across all four presented analyses (Colour figure online). (For interpretation of the references to colour in this figure legend, the reader is referred to the Web version of this article.)

Transcriptome profiling of males and females from the strains with deletions in *Gene Ontology* terms revealed dramatic changes in many cellular processes, including cellular response to oxidative stress, oxidation-reduction process, response to heat and, importantly, glutathione metabolic process (Fig. 1). Similarly, KEGG analysis (Fig. 2) revealed changes in glutathione, drugs and xenobiotic metabolism in females and, to a lesser degree, in males in the strains containing *CBS−/−* genotype. In the strains with the *CSE−/−* genotype, these alterations are less certain, while in the *MST−/−* strain, such changes were not detected. The most pronounced changes are observed in the whole bodies of *CBS−/−* flies, while in the thoraxes lacking ovaries, only xenobiotic metabolism is affected. We also detected clear-cut differences between males and females regarding changes in several important metabolic pathways, including carbon metabolism, biosynthesis of amino acids, glycolysis and pyruvate metabolism (Fig. 2). This finding can be explained by the gender gap in the ageing process and, specifically, by sex-dependent characteristic changes in metabolic profiles in the course of ageing described in fruit flies by different groups (see Ref. [38]).

**Fig. 3:**
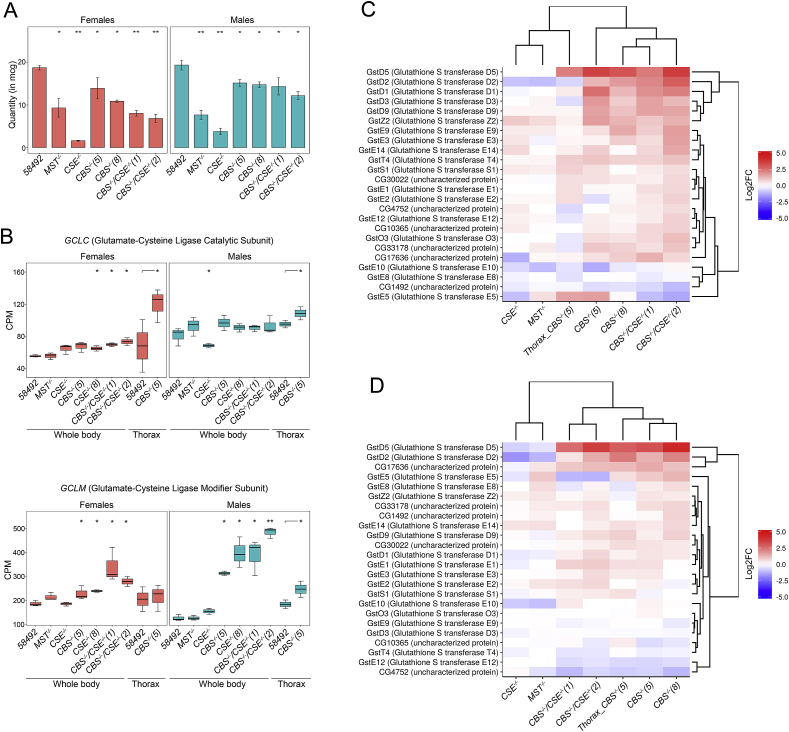
Effect of deletions on glutathione metaboloic process. A Spectrophotometric quantification of total nonprotein reduced thiol levels – mainly GSH. The GSH level in the whole body of males and females of control strain 58492 and *CBS−/−*, *CSE−/−*, *MST−/−,* (*CBS−/−*, *CSE−/−*) mutant flies was determined at 412 nm using a Lambda 25 spectrophotometer. Values are the means of 5 independent experiments. *P ≤ 0.05, **P ≤ 0.01. B. Box plots of *GCLC* (glutamate-cysteine ligase catalytic subunit) and *GCLM* (glutamate-cysteine ligase modifier subunit) expression levels in control strain (58492), *CBS−/−*, *CSE−/−*, *MST−/−* and (*CBS−/−*, *CS E−/−*) mutant flies (whole body); 58492 and *CBS−/−(5)* only thoraxes. CPM – counts per million; *P ≤ 0.05, **P ≤ 0.01. C – females, D – males. Heat map illustrating RNA-Seq differential expression data for *CBS−/−*, *CSE−/−*, *MST−/−* and (*CBS−/−*, *CSE−/−*) mutant flies (whole body); and *CBS−/−(5)* only thoraxes. Gene expression analysis for genes involved in the GO term GO0006749-glutathione metabolic process. Pairwise comparisons relative to the control strain are shown. Red positive log fold-change (log2FC). Blue, negative log2FC. . (For interpretation of the references to colour in this figure legend, the reader is referred to the Web version of this article.)

Most changes observed in *Gene Ontology* terms suggest that in the flies with deletions and, particularly, in *CBS−/−* containing strains, oxidative stress takes place, which results in the activation of several adaptive systems to prevent toxification (Fig. 1, Fig. 2). GSH represents one of the major cellular antioxidants [39]. To this end, our experiments exploring spectrophotometric determination of GSH (Fig. 3A) demonstrated that the level of total GSH is significantly reduced in the strains containing deletions of *CSE* and *MST* genes. In the flies with the *CBS−/−* genotype (single and double deletions), the level of GSH was also decreased in comparison with the control strain, but the decrease was less dramatic (Fig. 3A). Notably, in all strains with deletions, the observed decrease in GSH level was more pronounced in the females (Fig. 3A).

Glutamate-cysteine ligase, also known as γ-glutamyl cysteine synthetase, contains two subunits (Gclc and Gclm) that participate in the first limiting stage of GSH formation [40]. Characteristically, we observed a tendency towards increased *Gclc* expression in the males of most strains with deletions (with the exception of *CSE−/−*), while in the females, an enhanced level of *Gclc* expression was not revealed only in *MST−/−* flies (Fig. 3B). Notably, *CBS−/−* females, in contrast to males, exhibited a pronounced increase in *Gclc* expression detected only in thoraxes. The expression of a gene encoding another subunit (*Gclm*) was significantly elevated in the males of *CBS−/−* strains (Fig. 3B) and, to a lesser extent, in the females of *CBS−/−* strains. Most likely, the observed increase in the expression of these two genes represents a feedback compensatory response to the diminished amount of GSH resulting from the disruption of methionine metabolism taking place in the strains comprising the *CBS−/−* genotype. The higher content of GSH observed in flies comprising the *CBS−/−* genotype (Fig. 3A) is probably due to elevated activities of *Gclc* and *Gclm* genes observed in such flies to compensate for the severe disruption of methionine metabolism.

Another interesting feature of the strains containing *CBS* deletions, including the strains with double deletions, is an elevated level of transcription of multiple genes encoding glutathione transferases (GSTs) (Fig. 3C and D). These proteins are ubiquitous key enzymes that catalyse the conjugation of glutathione to various xenobiotic compounds and hence play a vital role in the detoxification process [41]. The deletion of *CBS* resulted in a dramatic increase in the transcription of most *Drosophila* GSTs in both sexes. Females exhibited a more pronounced increase in GST gene expression in comparison with males from the strains with *CBS* deletion (Fig. 3C), which is apparently due to a significantly lower level of GSTs in the females of the control strain (Figure S5). Notably, a similar pattern was observed in the strains with deletion of *CBS,* with the only prominent exception being the *GSTE8* gene, which exhibited a higher level of expression in the females (Figure S5). Importantly, the expression of individual GSTs is similar in the thoraxes of males and females.

In the strains containing *CSE* and *MST* deletions, we observed only slight changes in the levels of various GSTs in comparison with the control strain. Characteristically, only the level of *GSTZ2* transcription (Fig. 3C and D) was increased in all strains with deletions of the studied genes (especially in females). This gene of mitochondrial localization is expressed in the heads of adult flies. Females in the strains comprising the *CBS−/−* genotype exhibited enhanced levels of expression of *GSTD1*, *GSTD2*, *GSTD3*, *GSTD5*, *GSTD9*, *GSTE9* and *GSTZ2,* while in males, we observed a pronounced increase in *GSTD5*, *GSTD2*, *CG17636* and *Gclm*. Notably, the expression of *GSTE5* dropped in the *CSE−/−* and in strains with double deletion and increased in the flies with *MST* and *CBS* deletions (especially in males). In addition, in males and females with *MST* and *CSE* deletions, we revealed a decrease in the expression of *GSTD2* and *GSTE10*, while *GSTD5* was downregulated only in males of these strains.

**Fig. 4:**
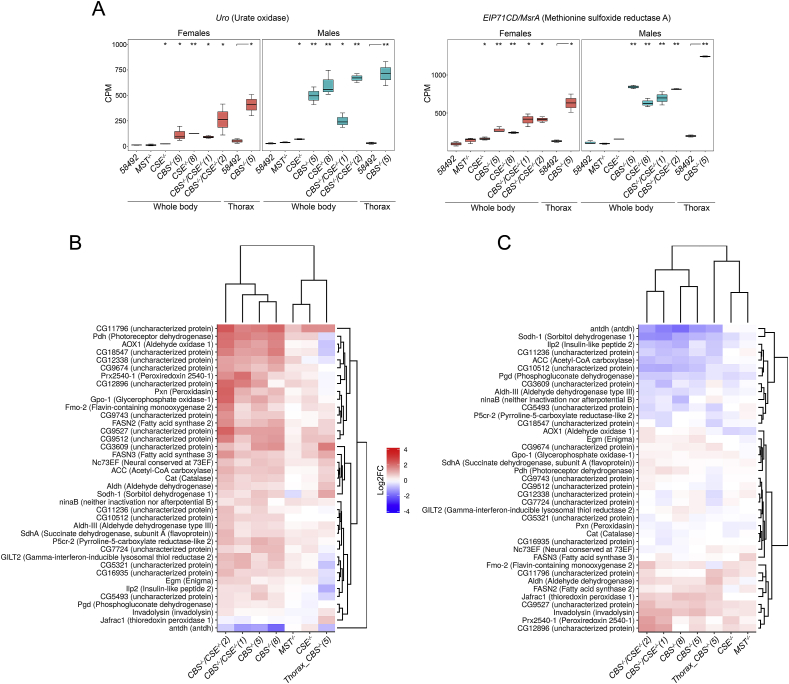
Effect of deletions on oxidation-reduction process. A. Box plots of *Uro* (Urate oxidase*)* and *EiP71CD* expression levels in control strain (58492), *CBS−/−*, *CSE−/−*, *MST−/−* and (*CBS−/−*, *CSE−/−*) mutant flies whole body; 58492 and *CBS−/−*(5) only thoraxes. CPM – counts per million; *P ≤ 0.05, **P ≤ 0.01. Heat map illustrating RNA-Seq differential expression data for *CBS−/−*, *CSE−/−*, *MST−/−* and (*CBS−/−*, *CSE−/−*) mutant flies whole body; and *CBS−/−(5)* only thoraxes Gene expression analysis for genes involved in Go term GO0055114 – oxidation-reduction process. B – females, C – males. Pairwise comparisons relative to the control strain are shown. Red positive log fold-change (log2FC). Blue, negative log2FC. (For interpretation of the references to colour in this figure legend, the reader is referred to the Web version of this article.)

**The compensatory response of diverse groups of oxidation-reduction genes is observed in the strains with deletions**. Multiple genes involved in the oxidation-reduction process represent another group that responded to the deletion of *CBS* and, to a lesser degree, of *CSE* and *MST* by transcription induction of its multiple members, including several oxidases, dehydrogenases, peroxidases, reductases, and cytochromes (Fig. 4). Characteristically, the maximal level of induction in the flies of both sexes in *CBS−/−* and *CSE−/−* strains was detected for the urate oxidase (*Uro*) gene expressed in the Malpighian tubules (Fig. 4A). The observed upregulation of *Uro* gene expression observed in the strains with deletions may also result from imbalance of purine metabolic process (see Fig. 2).

The methionine sulphoxide reductase gene (*EiP71CD*) is another locus activated in the males and females of all strains with deletions with the exception of males from *MST−/−* strains (Fig. 4A). This gene is involved in the cellular response to oxidative stress, sulphur amino acid metabolic process and determination of adult lifespan [42]. The reduction of oxidized methionine residues back into functional methionine restores the biological function of various proteins. Thus, expression of the *EiP71CD* gene plays an important role in the protein repair system and can reverse damage to proteins due to oxidation of methionine residues in proteins to methionine sulphoxide (met-(o)) [43,44].

Generally, the most dramatic increase in the expression of genes involved in the oxidation-reduction process was detected in the females from the strains containing *CBS* deletions. Notably, the expression level of most genes comprising this group was significantly lower in the females than in males in all strains (Figure S6).

Several genes of this group are upregulated in the females of all strains with deletions (i.e., *CG11796*, *CG9674*, *Pdh*, *Prx2540-1*, *Cg12896*, *SdhA*, and *P5cr-2*). In flies with *CSE−/−* and *CBS−/−* genotypes, *CG9527*, *CG9512*, *Gpo-1*, *CG3609*, and *FASN3* genes are upregulated. Interestingly, only one gene (*antdh*) was downregulated in strains with the *CBS−/−* genotype (Fig. 4B). The comparison of expression patterns of the considered group of genes between intact flies and females lacking ovaries and spermathecae (thoraxes) enables us to conclude that several of these genes are predominantly induced in these organs of the *CBS−/−* flies (i.e., *AOX1*, *Cg18547*, *CG12338*, *CG9674*, *Cg12896*, *Prx2540-1*, *Pdh*, *Pxn*, and *GPO-1*).

In the males but not the females of the *CBS−/−* genotype, a significant upregulation of the *jafrac* (thioredoxin peroxidase 1) gene plays an important role in the hydrogen peroxide catabolic process. In all strains with deletions, invadolysin and *CG9527* are upregulated, while *Aldh* does not change its expression only in the *MST−/−* strain. Surprisingly, the expression of several genes participating in the oxidation-reduction process, such as *Sodh-1*, *AldhШ*, *Pdh* and *Cg10512,* was downregulated in males with the *CBS−/−* genotype. Similarly, males with deleted *MST* and *CSE* genes exhibited downregulation of *AOX1* and *Pgd* genes (Fig. 4C).

**Fig. 5:**
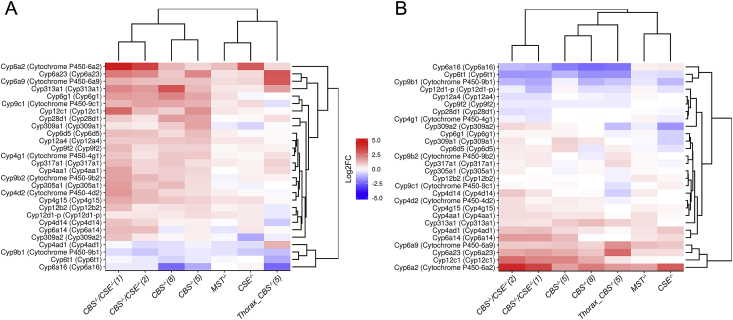
Effect of deletions on Cytochrome P450-mediated oxidation-reduction process. Heat map illustrating RNA-Seq differential expression data for *CBS−/−*, *CSE−/−*, *MST−/−,* (*CBS−/−*, *CSE−/−*) mutant flies (whole body); and *CBS−/−(5)* only thoraxes (A – females, B – males). Gene expression analysis for genes involved in the GO term GO0055114 - Cytochrome P450-mediated oxidation-reduction process. Pairwise comparisons relative to the control strain are shown. Red positive log fold-change (log2FC). Blue, negative log2FC. (For interpretation of the references to colour in this figure legend, the reader is referred to the Web version of this article.)

Cytochrome P450 enzymes also play an important protective role in the detoxification of foreign compounds and are known to catalyse a highly diverse range of chemical reactions that are important in both detoxification and in normal developmental processes. In the females of the control strain, the expression level of these genes was significantly lower than in males (Figure S7). Hence, we detected relatively higher induction of most of these genes in the females in the strains with deletions (Fig. 5A and B).

One of these genes (*Cyp6a2*) comprises an “antioxidant responsive element” (ARE) in its promoter. This gene is upregulated in all strains with deletions in both sexes, especially with the *CSE−/−* genotype. In females, most genes were predominantly upregulated in strains containing the *CBS−/−* genotype, including the strains with double deletions (Fig. 5A). Interestingly, the expression of three genes belonging to the cytochrome P450 group (*Cyp6a23*, *Cyp6a2*, and *Cyp6a9*) was upregulated in the thoraxes and whole body of the flies of all experimental strains with deletions. Strains *CBS−/−* and strains with double deletions were also characterized by a pronounced increase in the levels of *Cyp12с1*, *Cyp313a1* and *Cyp4aa1* expression. The majority of genes of this group exhibited a significant increase predominantly in the *CBS−/−* intact females, and their expression probably occurs in spermathecae, providing optimal conditions for sperm storage and functioning. In contrast, the level of *Cyp4ad1* expression is upregulated only in *CBS−/−* males (Fig. 5B). Notably, the expression of three cytochrome P450 genes (*Cyp6t1*, *Cyp6a16* and *Cyp9b1*) is downregulated predominantly in males with the *CBS−/−* genotype. Flies of both sexes with *the CSE−/−* genotype are downregulated genes *Cyp309a2* and *Cyp309a1* and, in males, *Cyp6g1* and *Cyp6d5*.

**Fig. 6:**
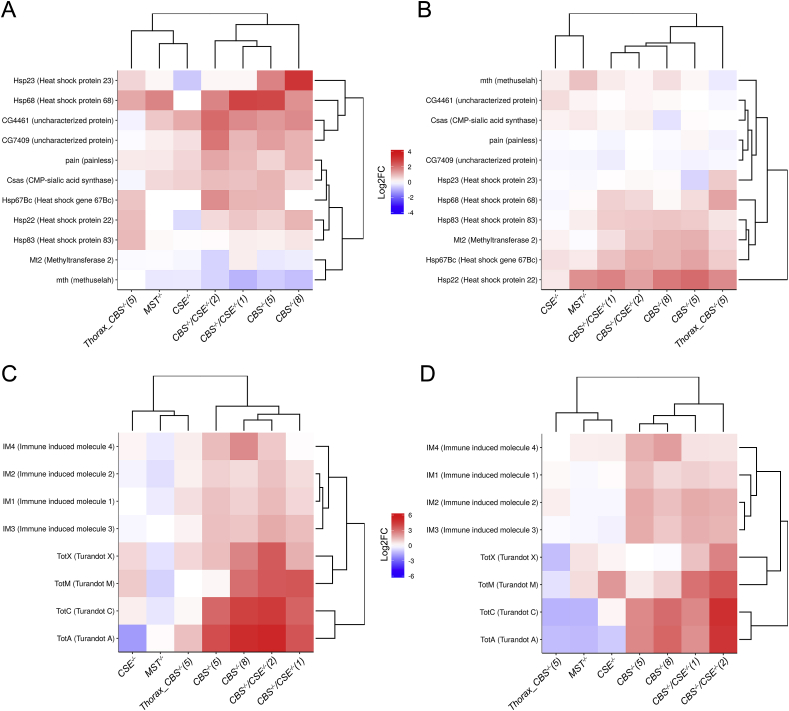
Effect of deletions on expression of stress-response genes. Heat map illustrating RNA-Seq differential expression data for *CBS−/−*, *CSE−/−*, *MST−/−,* and (*CBS−/−*, *CSE−/−*) double mutant flies (whole body); and *CBS−/−*(5) – thoraxes. Gene expression analysis for genes involved in the GO term GO00094 - response to heat. A, C – females. B, D – males. Pairwise comparisons relative to the control strain are shown. Red positive log fold-change (log2FC). Blue, negative log2FC. C and D heat map illustrating the expression of *Turandot* family genes and genes encoding *immune response molecules* that play important roles in humoural defence. (For interpretation of the references to colour in this figure legend, the reader is referred to the Web version of this article.)

**Expression of several stress-response systems is activated in strains with deletions**. The dysregulation of H_2_S production and consequent cellular oxidative stress apparently induces the activities of several stress-response systems that exist to combat this effect. Thus, several genes belonging to the heat shock gene system are upregulated in the strains with deletions. In this study, we observed induction of *hsp68* in the females of all strains lacking *CBS* and in the *MST−/−* strain but not in the strain comprising the deletion of *CSE*. It is known that the *hsp68* gene belongs to the Hsp70 family but exhibits characteristic differences in the structure of the regulatory region (i.e., GAGA sites are absent in the *hsp68* gene) [45]. In addition to *hsp68* induction, we observed a significant increase in the expression of *hsp22* in the males containing *CBS* and *MST* deletions (Fig. 6A and B). Interestingly, in contrast to *hsp68* in the case of *hsp22,* a strong induction was observed in the males, while females of these strains exhibited only slight induction of this gene transcription. Characteristically, in both sexes, the observed induction of *hsp22* takes place in the thoraxes but not in the gonads (Fig. 6A and B). There are a few other members belonging to the *hsps* group that exhibited slight but significant modulations in their expression in the strains with deletions of the three studied genes of sulphur metabolism (Fig. 6A and B). Interestingly, most of the above mentioned *hsps* genes are differentially expressed in males and females in the strains with deletions.

Surprisingly, the deletion of *CBS* significantly induced the expression of other unrelated groups of stress genes belonging to the *Turandot* family. In our analysis, we observed a manifold increase in the transcription of *Turandot A* and *C* and less *M* and *X* (*Tot*) in both males and females of the strains containing *CBS* deletion (Fig. 6C and D), while flies with CSE deletions exhibited only slight upregulation of *TotM* and *TotX* expression (Fig. 6C and D). As *TotA* and *TotC* represent maximal expression in the strains with *CBS* deletion in the strain with *CSE* knockdown, a drastic drop in the expression of *TotA* was demonstrated (Fig. 6C and D). Males of the strain with *MST* deletion exhibited a slight increase in the induction of *TotM* and *TotX* genes. Interestingly, *Drosophila TotA* was recently shown to encode a stress-induced humoural factor that gives increased resistance to the lethal effects of high temperature [46]. Along these lines, in the flies of both sexes comprising the *CBS−/−* genotype, significant induction of four genes encoding “Immune induced molecules 1–4” [47] that mediate the innate immune response was revealed (Fig. 6C and D).

**Response of diverse groups of housekeeping genes observed in the strains with deletions**. The deletion of the *CBS* gene exerted a maximal effect on the transcription pattern of the experimental strains in comparison with strains comprising the deletion of two other genes (*CSE* and *MST*) and apparently disturbed cellular homeostasis. The observed strong alterations in the expression of genes that participate in DNA repair processes corroborate this conclusion (Figure S8).). It is of note that in the control strain, the level of repair genes largely was significantly higher in females (Figure S9). In our analysis, we observed downregulation of several genes involved in repair in the *CBS−/−* flies and, in particular, the females containing double deletions, with the sole exception of the *eya* gene. This gene exhibited elevated expression in females with the *CBS−/−* and *CSE−/−* genotypes (Figure S8A). Gene “*eya*” represents a transcriptional cofactor that plays an important role in gamete generation, regulation of DNA repair and cellular processes involved in reproduction [48,49]. Furthermore, in females with double deletions, the expression of several genes (*rad9*, *rad17*, *rad60*, and *smc5*) (Figure S8B) involved in double-strand break repair via homologous recombination was also downregulated. Among these genes, *rad9* and *rad17* are also involved in the DNA damage checkpoint and mitotic DNA replication checkpoint, playing important roles in the process of oogenesis where the recombination process takes place [50,51]. In males with the *CBS−/−* genotype, we observed slight but significant upregulation in the expression of the following genes: *obelius*, *Psf2* and *CG32756* (Figure S8C).

**Fig. 7:**
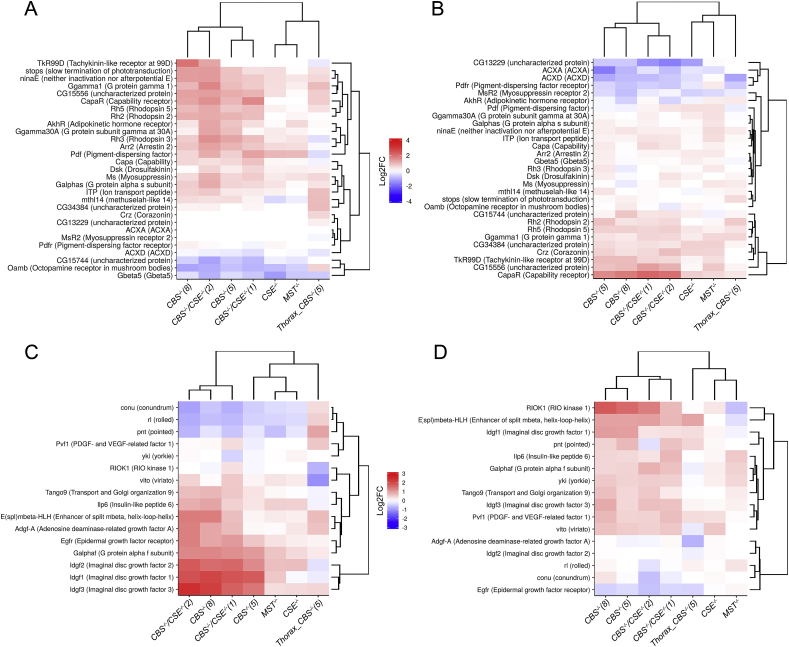
Effect of deletions on expression of genes involved in G protein-coupled receptor signaling pathway and in positive regulation of cell proliferation. Heat map illustrating RNA-Seq differential expression data for *CBS−/−*, *CSE−/−*, *MST−/−* and (*CBS−/−*, *CSE−/−*) mutant flies (whole body); and *CBS−/−*(5) only thoraxes. A - females, B – males. Gene expression analysis for genes involved in the GO term GO:0007186 - G protein coupled receptor signalling pathway. Gene expression analysis for genes involved in the GO term GO0008284 - positive regulation of cell proliferation. C - females, D - males. Pairwise comparisons relative to the control strain are shown. Red positive log fold-change (log2FC). Blue, negative log2FC. (For interpretation of the references to colour in this figure legend, the reader is referred to the Web version of this article.)

G protein-coupled receptor (GPCR) genes also belong to the housekeeping category and are known to be involved in the GPCR signal transduction system, which regulates many essential physiological processes in organisms [52]. Most of the genes belonging to the G-protein coupled receptor pathway were upregulated in the females and less upregulated in males from all strains comprising *CBS* deletion, while females from other strains with deletions (*CSE−/−*and *MST−/−*) exhibited only a tendency for induction (Fig. 7A and B). The performed analysis revealed another interesting feature: the activation of several genes in strains with *CBS−/−* and double deletions mostly in females (i.e., *nina E*, *arrestin 2*, *Rh2*, *Rh3*, *Rh5*) that participate in the phototransduction cascade and entrainment of the circadian clock. Additionally, in females of all strains, the expression level of the *Pdf* gene is upregulated. This gene is involved in the physiology of circadian rhythms [53]. In addition, significant upregulation of the *CapaR* and *Capa* genes is observed in males and females of the *CBC−/−* genotype. These genes operate in Malpighian tubules (MT) and regulate cellular ion and water balance after various stressful stimuli [54]. Interestingly, in male flies, genes encoding adenylyl cyclase genes (*ACXD* and *ACXA*) are downregulated. These genes play a role in the cAMP signalling pathway in *Drosophila* spermatogenesis [55].

Housekeeping genes involved in cell proliferation are also characteristically modulated in flies with deletions. Thus, mainly *CBS−/−* females exhibited a pronounced upregulation of the genes belonging to the *Idgf* family and several other factors participating in cellular proliferation, i.e., *Adgf-A* and *Ilp6* (Fig. 7C, D). On the other hand, females of all strains with deletions exhibited downregulation of several genes (i.e., *conu*, *rolled* and *pointed*) involved in proliferation. Notably, in males with the *CBS−/−* genotype, more genes were upregulated in this group than in females (Fig. 7C, D).

The deletions of the three genes participating in sulphur metabolism sometimes resulted in unexpected effects on the expression of genes. Thus, the deletions of these genes resulted in highly significant but diverse effects on the genes involved in mating behaviour and reproduction systems. Interestingly, in males and females with the *CBS−/−* genotype, a pronounced decrease in the expression level of genes encoding odour binding protein (*Obp*69a) and *lush* was observed. In *CBS−/−* females and males, we detected a decrease in the expression of the odour receptor coreceptor gene (*Orco*), which forms complexes with ligand-selective odorant receptors. In addition, in *CBS−/−* genotypes in males, a decrease in the expression of the gene encoding sensory neuron membrane protein 1 required for a normal olfactory response was observed [56]. Similarly, the deletion of *CBS, CSE* and *MST* results in a decrease in the expression level of the juvenile hormone esterase gene [57], which is necessary for robust male courtship behaviour and mating success. Surprisingly, in the *CBS−/−* females, significant induction of several genes (i.e., *tumorous testis, Esterase 6 and Quick-to-court*) known to play important roles in the reproductive organs of males was demonstrated. Similarly, in females containing *CBS* deletion, the decrease in the expression of gene *squash* was evident (Figure S10). Mutant (*squ*) females are sterile and show dorsoventral patterning defects during oogenesis [58]. However, the detailed analysis of these groups of genes is beyond the scope of the present study and will be summarized in a separate paper coupled with the data on the structure of the reproductive system and behaviour characteristics of the strains with deletions of sulphur metabolism genes (paper in preparation).

## 4 Discussion

The realization of the biological importance of H_2_S in the wide spectrum of model organisms and in humans is helping to elucidate the pathogenesis of various human diseases, including cancer and AD, and may pave the way for innovative therapeutic interventions and prophylactics [[6], [7], [8],[59], [60], [61]].

It was shown that patients with genetic defects in the TSP are characterized by high levels of homocysteine, low levels of GSH and increased incidence of age-related pathologies [4].

However, the molecular mechanisms underlying a wide spectrum of H_2_S effects at the cellular and organismal levels are far from fully understood. Unfortunately, the deletion or impairment of the *CBS* gene usually results in sterility or lethality of the organisms [62,63]. Therefore, the *D. melanogaster* strains developed in our laboratory containing single and double deletions of the three major genes involved in the regulation of H_2_S production represent a unique tool to identify physiological functions of the hydrogen sulphide and pinpoint molecular pathways in the cells participating in the interaction with this important signal molecule. H_2_S elicits cytoprotection during oxidative stress by decreasing ROS production in a wide range of physiological and pathological conditions [64,65]. Our analysis of genome-wide transcriptional consequences of the deletions of major sulphur metabolism genes in fruit flies enabled us to define various adaptive systems and signal pathways that apparently interact with H_2_S and are activated in flies with deletions (Fig. 1, Fig. 2).

The detected pronounced modulations in the functioning of several vital systems of the fruit flies (Fig. 1, Fig. 2) indicate the occurrence of compensatory and apparently adaptive responses to the disturbed metabolism of methionine and H_2_S production observed in the flies with deletions. It is known that adaptive responses may make an organism resistant to various forms of stressful stimuli [[66], [67], [68], [69], [70], [71], [72]]. Along these lines, higher resistance of all strains with deletions to superoxide producer paraquat was demonstrated (unpublished data). Characteristically, in our transcriptomic analysis, the most striking changes were found in the flies containing the deletion of *CBS* (single or double, i.e., in combination with *CSE−/−*). Single *CSE* and *MST* deletions result in milder changes. Notably, we failed to observe significant compensation in the expression of individual studied genes involved in H_2_S production in the strains with deletions. The increased expression of *CSE* observed in the males of the *CBS*−/− strain was a prominent exception (Figure S3, S4).

The deletion of *CBS* and, in particular, double deletion of both genes representing major H_2_S-producing loci apparently results in oxidative stress. As a consequence, this stress induces various genes and signalling pathways participating in antioxidant defences and detoxification. In the strains with deletions, we observed dramatic modulation of the genes involved in the basic oxidation–reduction reaction, which operates in all cells and is vital for cell homeostasis and survival of an organism (Fig. 1, Fig. 2, Fig. 3, Fig. 4 and 5). The obtained results are expected because H_2_S exercised its protective effects in part by inhibiting mitochondrial electron transport and oxidative phosphorylation, leading to increased glucose uptake and glycolytic ATP production [7].

Antioxidant defences include catalases, peroxidases, superoxide dismutases (SOD), and glutathione S-transferases (GST). Antioxidant-specific gene induction, involved in xenobiotic metabolism, is mediated by the “antioxidant responsive element” (ARE). ARE is found in promotors of GSTs, P-450 genes, SOD, catalases (CAT) and peroxidases. Many genes upregulated in *CBS−/−* flies encode P450s, glutathione S-transferases (GST) and esterases and play important roles in detoxification and insecticide resistance [[73], [74], [75]].

Another interesting group of genes strongly induced by *CBS* deletion consists of genes involved in glutathione metabolic processes (Fig. 3) [40,41,76].

GSH is a downstream metabolite of TSP, and its synthesis is dependent on the availability of cysteine. Furthermore, cysteine availability controls the synthesis of glutathione (GSH), which is the major regulator of cellular redox homeostasis. In our investigation, we demonstrated a drastic drop in GSH in lines with *CSE* and *MST* deletions (Fig. 3A). Furthermore, flies with the *CBS−/−* genotype exhibited significantly higher levels of GSH than flies with other deletions (*CSE* and *MST*). This characteristic feature probably resembles more severe oxidative stress due to disruption of methionine and GSH metabolism in such flies and increased compensatory activity of several genes involved in GSH synthesis and metabolism (Fig. 3B).

Notably, GSH levels were increased in diet-restricted flies, and the cellular level of methionine has been implicated in murine ageing [4]. It is known that several enzymes participate in GSH redox homeostasis, including glutathione peroxidase (*Prx*), glutaredoxin (*Grx*), and thioredoxin (*Trx*). In our transcriptomic analysis, we demonstrated increased expression of the peroxiredoxin 2540-1 gene in males and females with the *CBS−/−* genotype, while females with *CBS* deletion exhibited enhanced expression of the glutaredoxin 1 (*Grx1*) gene. Furthermore, *CBS−/−* males were characterized by a significant upregulation of the thioredoxin peroxidase 1 gene *jafrac* (Fig. 4). Our analysis also revealed a strong modulation of GST expression in flies with deletions (Fig. 3). GSTs localized in the cytoplasm are involved in glutathione metabolic processes. At the present time, 40 GSTs were identified in *Drosophila melanogaster*, and the Delta and Epsilon groups of these enzymes are insect-specific and apparently function in detoxification and insecticide resistance [76]. Protein glutathionylation can also protect proteins from oxidative states and modulate their activity [77,78]. Thus, direct interaction between GST and mitogen-activated protein kinase (p38) activates this protein and exhibits anti-inflammatory effects [78]. On the other hand, GAPDH is inactivated after oxidative stress via glutathionylation on Cys149 in endothelial cells [79].

An important and unexpected feature revealed in the course of our transcriptomic analysis is the strikingly different response of males and females to the deletion of the genes involved in methionine metabolism and H_2_S production and, in particular, to *CBS* deletion. Striking gender differences were detected in all of the above-described systems (i.e., glutathione metabolic process, oxidation-reduction process, cytochrome P450-mediated oxidation-reduction process, and DNA repair).

To this end, we demonstrated significant sex-dependent changes in cytochrome P450 gene expression in all strains with deletions (Fig. 5). Sex dependence of P450 induction was also found in other investigations [80]. Several enzymes belonging to the P450 group were shown to play key roles in the metabolism or activation of xenobiotics [81]. Most of these enzymes (**FlyAtlas Anatomical Expression Data**) are expressed predominantly in the midgut, hindgut, Malpighian tubule head and spermatheca, where the metabolism of both exogenous and endogenous compounds takes place. Importantly, these tissues are involved in detoxification processes and protection from harmful exogenous compounds. Thus, it was recently demonstrated that the Malpighian tubules are important for the metabolism and detoxification of xenobiotics and might also be involved in immunity [72,82]. Exposure of *Drosophila* to toxins evokes a coordinated response by the Malpighian tubules (MT), involving changes in detoxification systems and enhanced transport [83]. Similarly, in our studies, we demonstrated modulation in the expression of multiple genes participating in MT function. The induced expression of the *Uro* gene in *CBS−/−* flies represents a typical example of such regulation (Fig. 4A). This gene is usually upregulated in the case of inflammation induced by the accumulation of uric acid crystals in cells [84]. It is known that the disturbance in the CBS/CSE system results in homocystinuria and the development of inflammation in the excretory system in mammals [63]. The observed upregulation of *Uro* gene expression observed in the strains with deletions may also result from imbalance of purine metabolic process (Fig. 2).

In our studies, we also demonstrated a significant upregulation of *CapaR* and *Capa* genes (Fig. 7A and B) observed in males and females of the *CBC−/−* genotype. The *Capa* gene in *Drosophila* encodes neuropeptides, while *CapaR* is responsible for the synthesis of receptors for these neuropeptides. Specifically, *Capa* acts as a diuretic hormone in the Malpighian tubules to regulate cellular ion and water homeostasis after desiccation and stress [54]. There are other data corroborating our conclusion concerning the increased excretory function of MT in flies with deletions. Thus, in all lines with deletions, we detected upregulation of genes encoding proteins with inwardly rectifying potassium channel activity (Irk3). In addition, in flies with the *CBS−/−* genotype, upregulation of Irk2 and salty dog (*salt*) genes playing important roles in MT function [85,86] was detected (Figure S11).

Our transcriptomic analysis revealed significant changes in the activity of a notably ancient and conserved system of heat shock genes induced by numerous agents [87,88]. Heat shock genes encode proteins with different molecular weights and functions generally involved in proteostasis under normal and stressful conditions in all eukaryotic organisms [89]. There are scattered data suggesting some cross-talk between the genes participating in methionine metabolism and *hsps* [19,90,91].

In our analysis, we observed a significant increase in the expression of *hsp22* in the males containing *CBS* or *MST* deletion (Fig. 6). Hsp22 is a well-known beneficial protein because its overexpression increases lifespan and resistance to stress, while its downregulation is detrimental [[92], [93], [94], [95], [96]]. Interestingly, in contrast to *hsp68* in the case of *hsp22,* a strong induction was observed in the males, while females of these strains exhibited only slight induction of this gene transcription. Characteristically, in both sexes, the observed induction of *hsp22* takes place in the thoraxes but not in the gonads (Fig. 6). There are several other members belonging to the *hsps* group that exhibited slight changes in their transcription in the strains with deletions of the three studied genes of sulphur metabolism (Fig. 6A and B).

Surprisingly, the deletion of *CBS* significantly induced the expression of another unrelated family of stress genes belonging to the Turandot family (Fig. 6C and D). The induction of the *Tot* genes differs in important respects from the heat shock response, such as the strong but delayed response to bacterial infection demonstrated for several of the genes. Additionally, these genes can be induced by heavy heat shock, oxidative stress, dehydration and UV irradiation [46,97]. Interestingly, *Drosophila TotA* was recently shown to encode a stress-induced humoural factor that gives increased resistance to the lethal effects of high temperature [97].

There are other unrelated gene families providing survival under various stressful conditions that were upregulated in the flies with deletions, apparently as a compensatory response to the disturbed sulphur metabolism. Thus, *CBS−/−* flies, especially females, exhibited a pronounced upregulation of the genes belonging to the *Idgf* family (Fig. 7C and D). These genes are structurally related to chitinases and have acquired a new growth-promoting function [98]. One of the upregulated genes in the *CBS−/−* female gene *Idgf2* belongs to this family and encodes a secreted glycoprotein mainly expressed in the fat body. The expression of *Idgf2* is induced under various stress conditions. The product of this gene is a trophic factor involved in energy balance, detoxification, and innate immunity [99]. Notably, genes belonging to the *Idgf* family, as well as two other (*Adgf-A* and *Ilp6*) genes, are upregulated in *CBS−/−* females and are involved in the regulation of cellular proliferation.

Since the expression of *CBS* and other genes producing H_2_S was shown to play an important role in the reproductive system of various organisms in several cases, we compared their level of expression in intact flies with corresponding levels in thoraxes with excised ovaries and spermathecae. These experiments demonstrated that in many cases, a high level of expression observed in females takes place predominantly in the ovaries or/and spermathecae (Fig. 3, Fig. 4, Fig. 5).

It is necessary to keep in mind that control males are characterized by significantly higher original levels of expression of most studied systems modulated in the strains with deletions (i.e., Figures S5, 6, and 7). Therefore, more dramatic modulations detected in the females from the strains with deletions are explained by the original gender differences in the studied gene systems.

Interestingly, we revealed severe defects in reproductive system development and functioning occurring in females lacking the *CBS* gene (unpublished data). The accumulated data enable us to conclude that the studied genes of methionine metabolism exercise different roles in the ontogenesis and reproduction of *Drosophila* males and females that respond to their deletion at the level of transcription in a sex-specific manner. We are well aware that the observed strong and sometimes unexpected effects of the deletions may be secondary to the well-known reducing activity of H_2_S and/or its ability to participate in sulphhydration of protein cysteine moieties in the cells.

To the best of our knowledge, this report is the first to describe the effects at the transcription level of single and double deletions of the three major genes responsible for H_2_S production in fruit flies. The ubiquitous membrane permeability characteristic of H_2_S and its unique chemical reactivity with various types of molecules makes H_2_S a selective and powerful signalling molecule. Our studies of genome-wide transcriptional consequences of the deletions of major sulphur metabolism genes in *D. melanogaster* reported in this study demonstrated the important role of these genes in such different aspects of organism biology in response to oxidative stress, as well as mating behaviour and reproduction.

## Funding

This work has been supported by Grant of 10.13039/501100006769Russian Science Foundation №17-74-30030 and Russian grants Program for Basic Science №19-04-00109 (to O.Z.) and №18-29-07015 (to D.G).

## Declaration of competing interest

The authors declare that they do not have any conflicts of interests.
