# Supplementary material for: Genome-wide transcriptional effects of deletions of sulphur metabolism genes in Drosophila melanogaster
Source: Redox Biol. 2020 Jul 25;36:101654. doi: 10.1016/j.redox.2020.101654 (PMC7414014; doi:10.1016/j.redox.2020.101654)
Supplement: Multimedia component 1 [file mmc1.zip › Table S1.docx]

**Table S1. Oligonucleotides used in CRISPR experiments**

| Name | Sequence (5'-> 3') |
| --- | --- |
| CSE_CG5345-cyto-5'-flank-genome-check-F | CAGCTATCCGTCTCTGAAC |
| 5'-flank-check-R | CAGGCAGCGAGATCATCAAG |
| CSE_CG5345-cyto-5'-flank-XbaI-F | GTATAATTTTCTAGACAAGGAGATCGTGTTCAGC |
| CSE_CG5345-cyto-5'_ex1-white-4_in-F | CCGTTTGATCCGACAGGTGAGTGGTTCCAGTGG |
| CSE_CG5345-cyto-5'_ex1-white-4_in-R | TGGAACCACTCACCTGTCGGATCAAACGG |
| CSE_CG5345-cyto-5'-white-4_in-XhoI-R | TTCTTCCTTCTCGAGAAGGGTGTGGAATCAGGCA |
| CSE_CG5345-3'-EagI-flank-F | TTCTTCCTTCGGCCGCTGCATTACCTCCACGAC |
| CSE_CG5345-3'-SacI-flank-R | CTTCTCCTTGAGCTCGCTTGGCTATCTAATGGG |
| CSE_CG5345_cyto-3'-flank-genome-check-R | CAAGGTGAGATAGCTAATGAG |
| 3'-flank-check-F | TCACGTTTTCCCAGGTCAGA |
| CSE_CG5345_cyto-5'-flank-sgRNA-F | TATATAGGAAAGATATCCGGGTGAACTTCGGCTGAGATTCCCAGACGGGGTTTTAGAGCTAGAAATAGCAAG |
| CSE_CG5345_cyto-3'-flank-sgRNA-R | ATTTTAACTTGCTATTTCTAGCTCTAAAACCATCTTATCAGTTCGCCACACGACGTTAAATTGAAAATAGGTC |
| MST_CG12279-5'-flank-genome-check-F | CACCGTATGACCGACCGTA |
| MST_CG12279-3'-flank-genome-check-R | GCTCTGTTGGAGGTGATGA |
| MST_CG12279-5'-flank-XbaI | CTCTTCTTTTCTAGACACACACCGTATCACCGTATG |
| MST_CG12279-5'-flank-XhoI-R | TTCTCTTCTCTCGAGCGGTAATCCCTGGACTTTG |
| MST_CG12279-3'-flank-EagI-F | ATCTCACGTCTCCGGCCGCTAATTTCCAGTGAGCGAGCTGGAGAAGG |
| MST_CG12279-3'-flank-SacI-R | CTAATCTCCGAGCTCATGTCTCAGGATAAGGCTCT |
| sgRNA-MST-’5-ol-F | TTCGTCCAGGGATTACCGGCTTAA |
| sgRNA-MST-’5-ol-R | AAACTTAAGCCGGTAATCCCTGGA |
| Primers for qRT PCR |  |
| rp49 F | 5’-ATGCTAAGCTGTCGCACAAAT-3’ |
| rp49 R | 5’-GTTCGATCCGTAACCGATGT-3’ |
| cbs 5newF | 5’-GCCAGCAGATTACGCCCAACA-3’ |
| cbs 5_R | 5’-TAAGGAACTCGCACTTGGCAT-3’ |
| cse F6 | 5’-CAACACCTTCCTGACCTCCTA-3’ |
| cse R6 | 5’-CGAATGGAGACGGCACAATG-3’ |
| mst F_3 | 5’-GGAGGAGGACTTCGCCCAG-3’ |
| mst R_2 | 5’-ATCGGACGCAGTTGGGGTG-3’ |
