# Supplementary material for: Genome-wide transcriptional effects of deletions of sulphur metabolism genes in Drosophila melanogaster
Source: Redox Biol. 2020 Jul 25;36:101654. doi: 10.1016/j.redox.2020.101654 (PMC7414014; doi:10.1016/j.redox.2020.101654)
Supplement: Multimedia component 1 [file mmc1.zip › Supplementary Materials.docx]

**Supplementary Materials.**

**The development of double deletions**. Two lines with double deletions were generated by crossing two independent cbs -/- lines (*cbs*5 and *cbs*8) and a line with *cse* deletion (*cse*1). Besides, two balancer lines: yw; CyO/If (chromosome 2), and ♀ Df(1)260-1, y[1]/FM4 (X-chromosome) were used. The double homozygotes (cbs-/- and cse-/-) were obtained by five following crosses:

P1 ♂ yw; CyO/If x ♀ CBS /CBS

P2 ♂ yw/CBS; CyO x♀ Df(1)260-1, y[1]/FM4

P3 ♀FM4/CBS; CyO x♂CSE/CSE

P4 ♂CBS; CyO /CSE x ♀ FM4; CyO /CSE

P5 ♀ FM4/CBS;CSE x ♂ CBS; CyO /CSE

After selection the obtained flies with double deletions were verified by Southern blotting and RTPCR as described in M&M.

**Figure legends to Supplementary Figures**

**Suppl. Figure 1.** Southern blot analysis to detect off-targets in flies with deleted *CBS*, *CSE* or *MST* genes. **A.** Genomic DNA from flies with deleted *CBS* or *CSE* genes was digested with *Bam*HI/*Pst*I or *Bam*HI restriction endonucleases, respectively: 1-4 – *CBS-/-* strains, 5-6 – *CSE-/-* strains; **B.** Genomic DNA from flies with deleted *MST* gene was digested with *Eco*RI restriction endonuclease: 1-9 – *MST-/-* strains, 10 – control strain 58492. Hybridization with the ^32^P-labelled *mCherry* gene. Off-targets marked by arrows.

**Suppl. Figure 2.** Southern blot analysis using flies with double deletion (*CBS* and *CSE* genes). Genomic DNA from flies with double deletion was digested with *Nco*I/*Pvu*I restriction endonucleases and hybridized with *CSE* ^32^P-labelled probe; *Cla*I restricted DNA was hybridized with *CBS* ^32^P-labelled probe. 1-10 – double deletion strains, 11 – control 58492 strain, 12-13 – *CBS-/-* strains, 14 – *CSE-/-* strain. **A** - hybridization with *CBS* ^32^P-zone, **B** - hybridization with *CSE* ^32^P-labelled zone.

**Suppl. Figure 3.** Validation of *CBS*, *CSE* and *MST* gene deletions mediated by the CRISPR/Cas9 system using quantitative RT-PCR. *MST-/-*, *CSE-/-*, and *CBS-/-* represent transgenic *Drosophil*a strains with knockout genes (*MST*, *CSE* and CBS, respectively); (*CBS-/-*, *CSE-/-*) represents two transgenic strains with the double deletion (*CBS* and *CSE*); 58492 is a control *Drosophil*a strain. *CBS* gene expression is absent in *CBS-/-* transformants in both males and females, and *CSE* gene expression was absent in *CSE-/-* transformants. Trace amounts of *MST* expression were detected in *MST-/-* transformants. Bars represent the average error for two independent experiments; the mean ± SD of the three technical replicates is shown. * *P* ≤ 0.05.

**Suppl. Figure 4.** Box-plots of *CBS* (Cystathionine β-synthase), *CSE* (Cystathionine gamma-lyase) and *MST* (3 mercaptopyruvate sulphurtransferase) expression levels in the control stock 58492 and (*CBS-/-*), (*CSE-/-*), (*MST-/-*)*,* (*CBS-/-*, *CSE-/-*) flies (whole body); 58492 and *CBS-/-*(5) only thoraxes. CPM – counts per million; * *P* ≤ 0.05, ** *P* ≤ 0.01.

**Suppl. Figure 5. Expression profile of genes implicated in Glutathione metabolic process.** The expression levels of all analysed female and male samples were normalized by Z-score. Clusterization performed using Canberra distance.

**Suppl. Figure 6. Expression profile of genes implicated in the oxidation-reduction process.** The expression levels of all analysed female and male samples were normalized by Z-score. Clusterization performed using Canberra distance.

**Suppl. Figure 7. Expression profile of cytochrome genes.** The expression levels of all analysed female and male samples were normalized by Z-score. Clusterization performed using Canberra distance

**Suppl. Figure 8.** **A** - Box plots of *eya* (eyes absent) and *EiP71CD* expression levels in control strain (58492) and *CBS-/-*, *CSE-/-*, *MST-/-* and (*CBS-/-*, *CSE-/-*) mutant flies (whole body); 58492 and *CBS-/-*(5) only thoraxes. CPM – counts per million; * *P* ≤ 0.05, ** *P* ≤ 0.01. **B** - females, **C** - males. Heat map illustrating RNA-Seq differential expression data for *CBS-/-*, *CSE-/-*, *MST-/-* and (*CBS-/-*, *CSE-/-*) mutant flies (whole body); and *CBS-/-*(5) only thoraxes. Gene expression analysis for genes involved in GO term GO0006281 - DNA repair. Pairwise comparisons relative to the control line are shown. Red positive log fold-change (log2FC). Blue, negative log2FC

**Suppl. Figure 9. Expression profile of genes implicated in DNA repair.** The expression levels of all analysed female and male samples were normalized by Z-score. Clusterization performed using Canberra distance.

**Suppl. Figure 10.** . Heat map illustrating RNA-Seq differential expression data for *CBS-/-*, *CSE-/-*, *MST-/-* and (*CBS-/-*, *CSE-/-*) mutant flies (whole body); and *CBS-/-*(5) only thoraxes. Gene expression analysis for genes involved in sexual reproduction and response to pheromone. **A** - females, **B** – males. Pairwise comparisons relative to the control strain are shown. Red positive log fold-change (log2FC). Blue, negative log2FC.

**Suppl. Figure 11.** Box-plots for *Irk2* (Inwardly rectifying potassium channel 2), Irk3 (Inwardly rectifying potassium channel 3), *salt* (salty dog) expression levels in the control stock 58492 and (*CBS-/-*), (*CSE-/-*), (*MST-/-*)*,* (*CBS-/-*, *CSE-/-*) flies (whole body); 58492 and *CBS-/-(5)* only thoraxes. CPM – counts per million; * *P* ≤ 0.05, ** *P* ≤ 0.01.
