# Supplementary figures and images for: Genome-wide transcriptional effects of deletions of sulphur metabolism genes in Drosophila melanogaster
Source: Redox Biol. 2020 Jul 25;36:101654. doi: 10.1016/j.redox.2020.101654 (PMC7414014; doi:10.1016/j.redox.2020.101654)

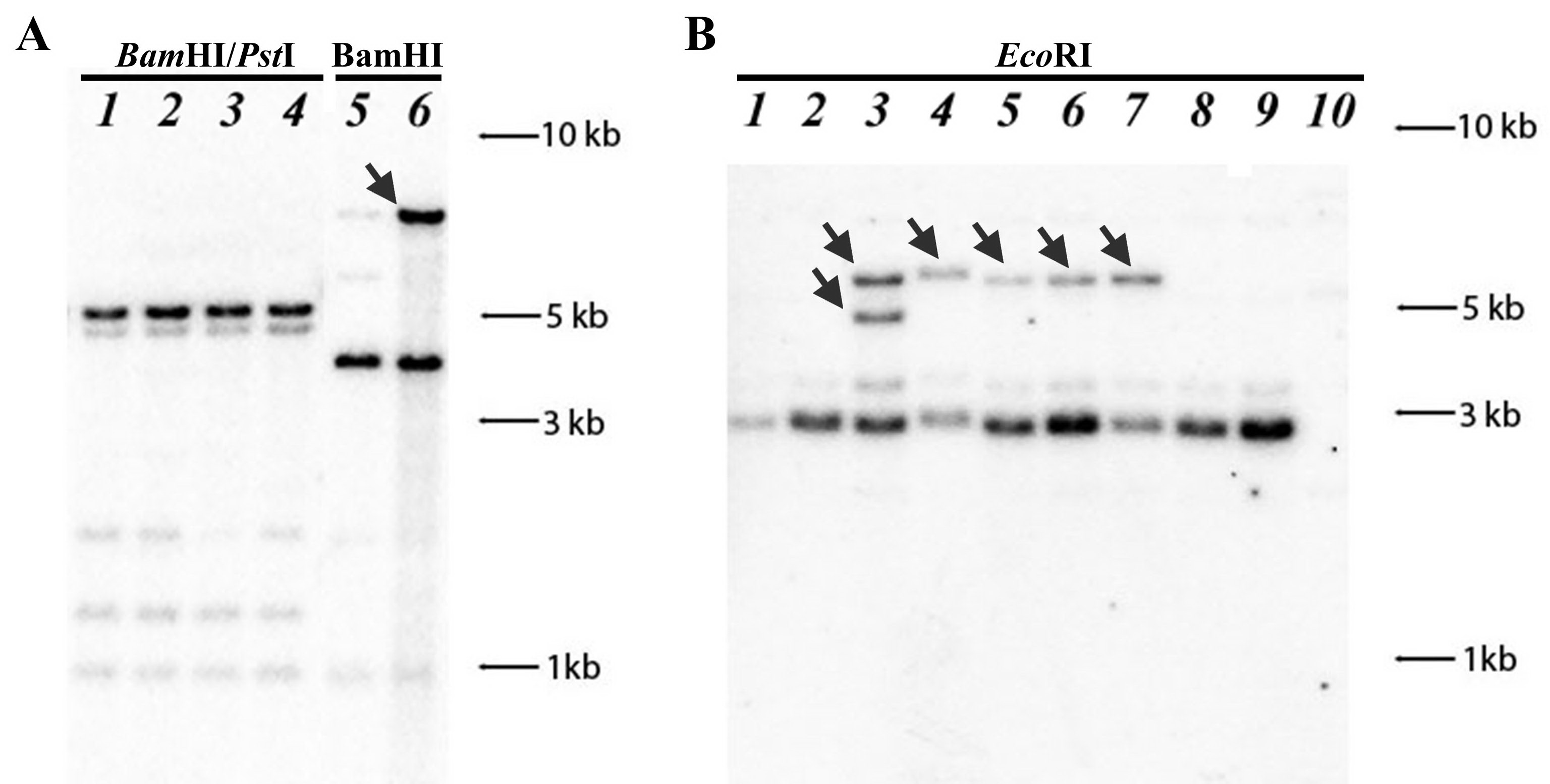

Supplement: Multimedia component 1 [file mmc1.zip › Figure_S1.tif]

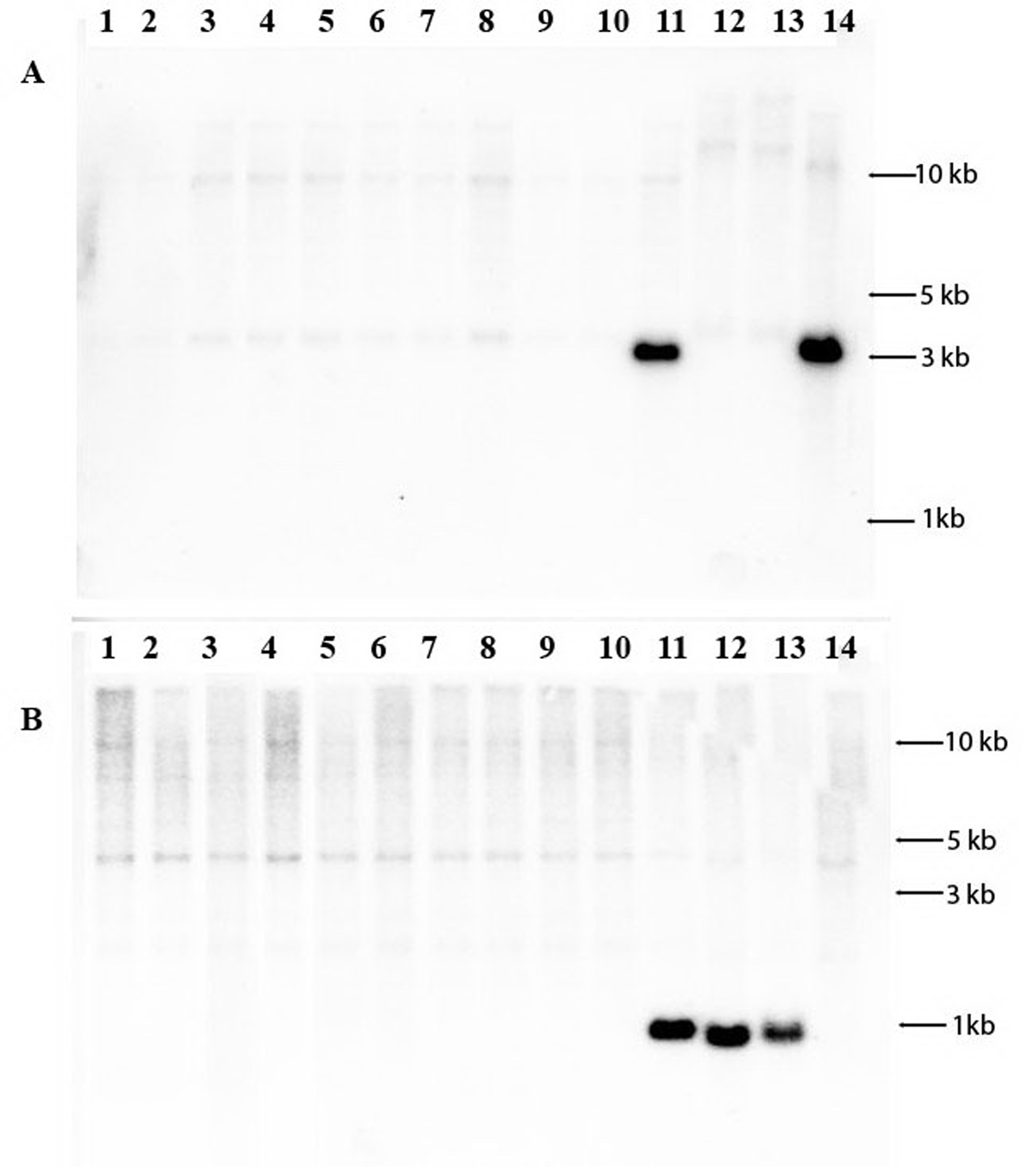

Supplement: Multimedia component 1 [file mmc1.zip › FIgure_S2.tif]

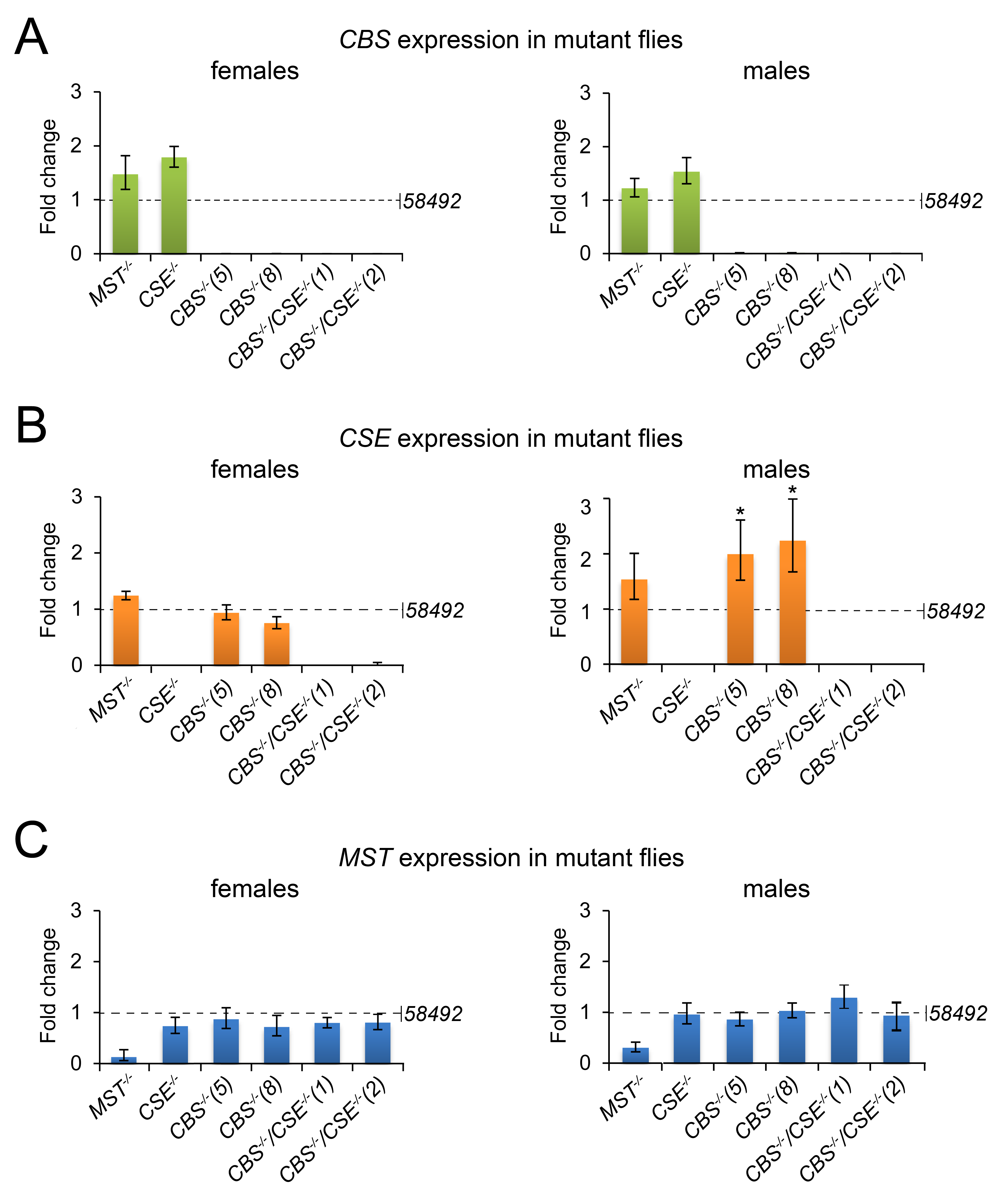

Supplement: Multimedia component 1 [file mmc1.zip › Figure_S3.tif]

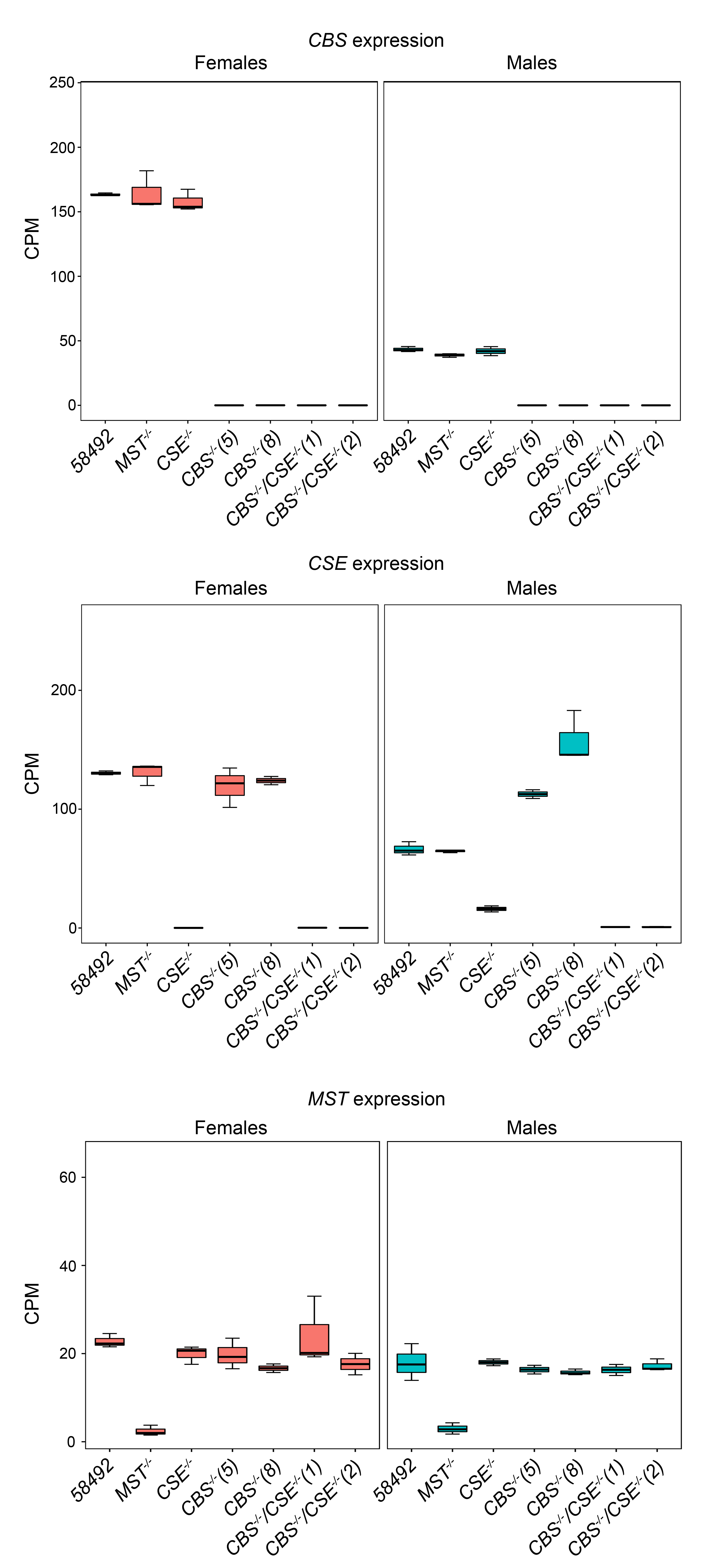

Supplement: Multimedia component 1 [file mmc1.zip › Figure_S4.tif]

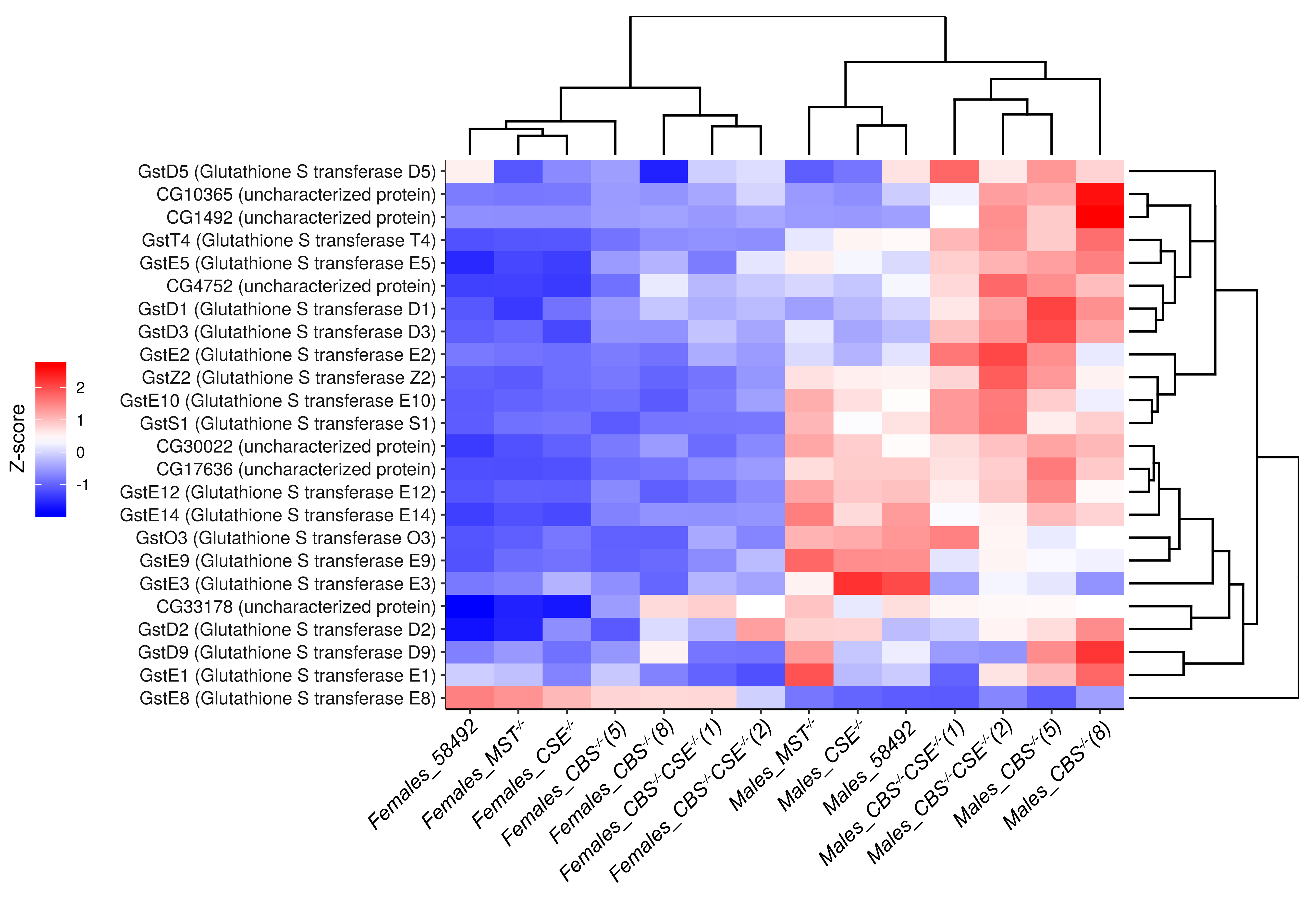

Supplement: Multimedia component 1 [file mmc1.zip › FIgure_S5.tif]

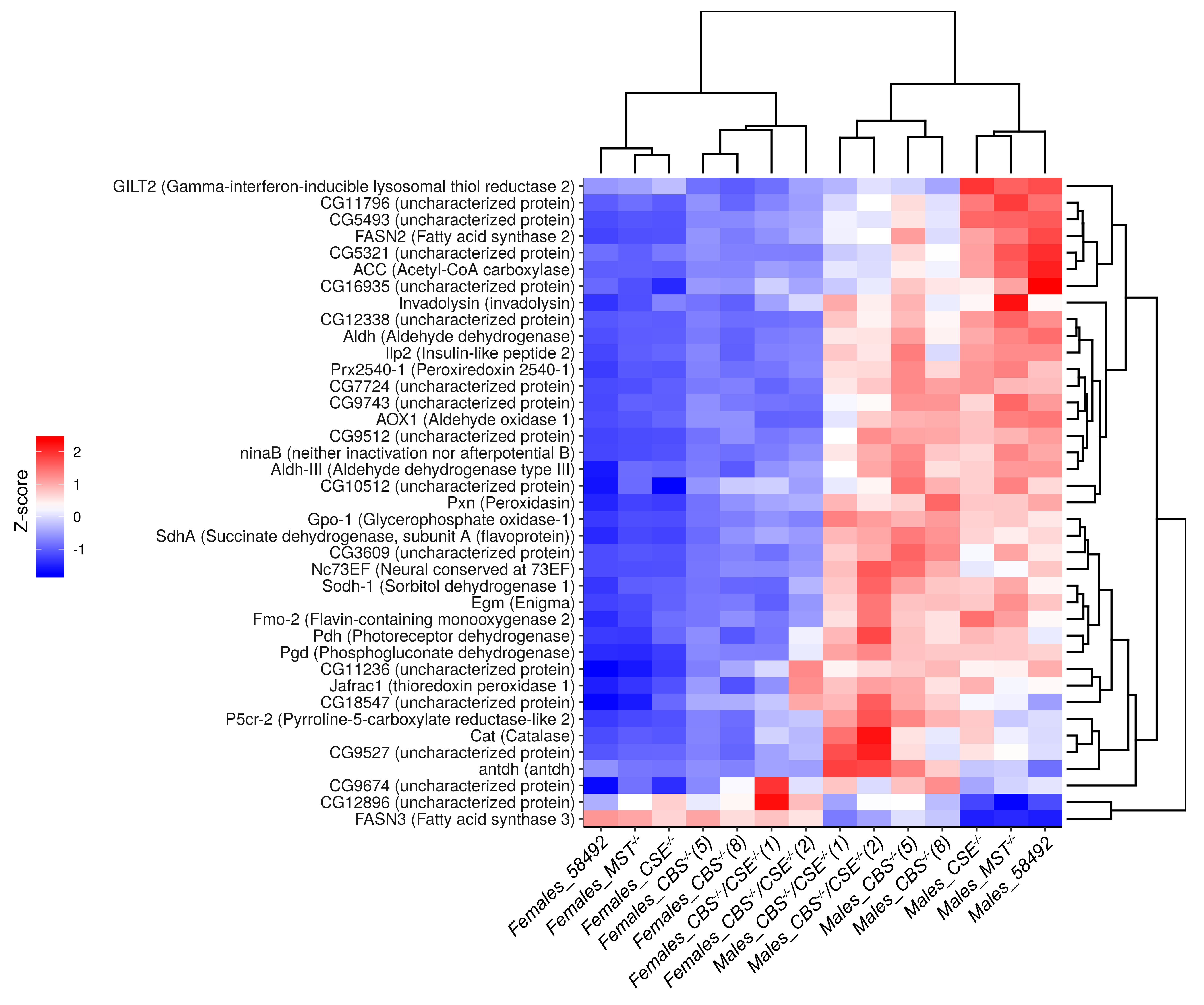

Supplement: Multimedia component 1 [file mmc1.zip › Figure_S6.tif]

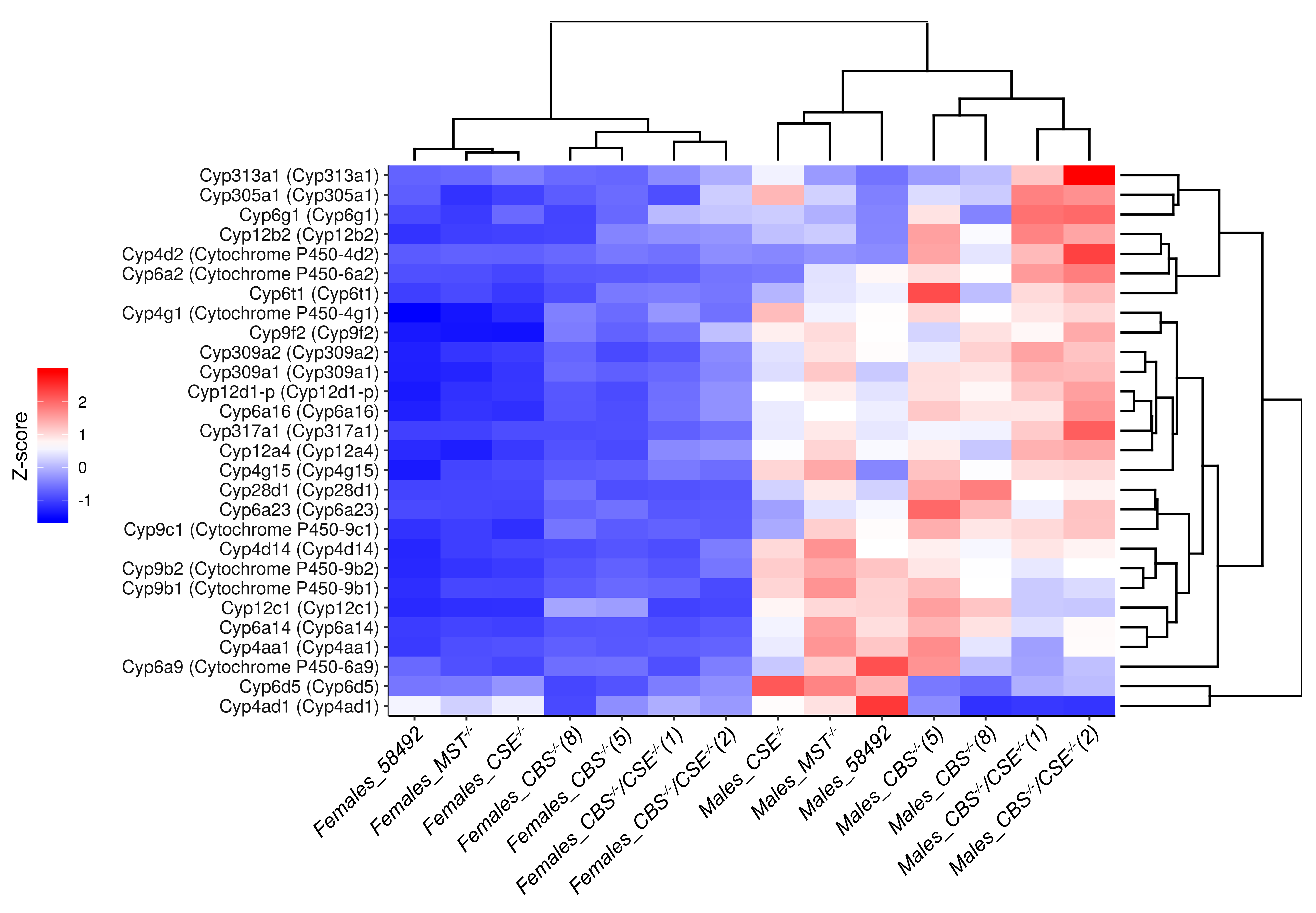

Supplement: Multimedia component 1 [file mmc1.zip › Figure_S7.tif]

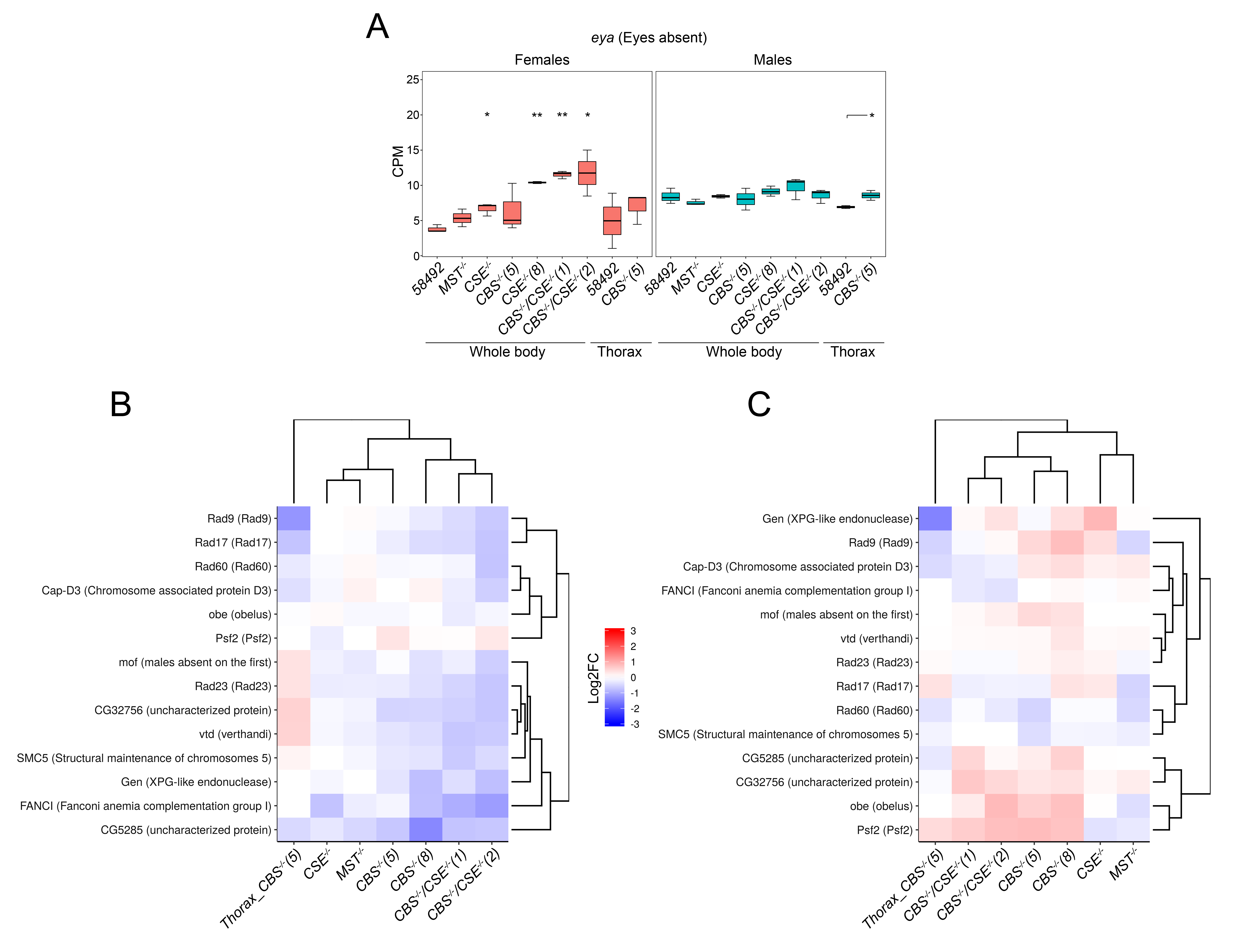

Supplement: Multimedia component 1 [file mmc1.zip › Figure_S8.tif]

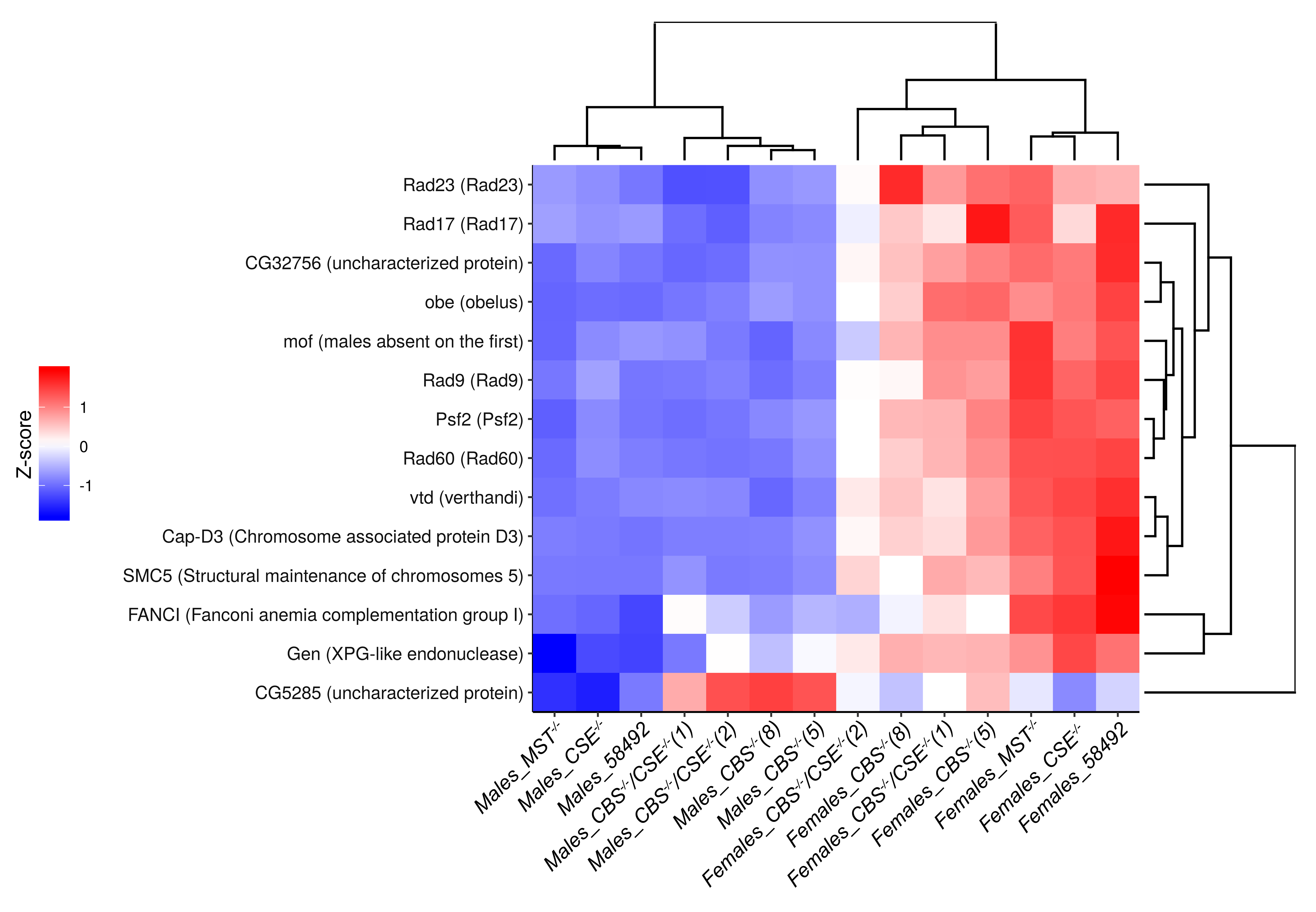

Supplement: Multimedia component 1 [file mmc1.zip › Figure_S9.tif]

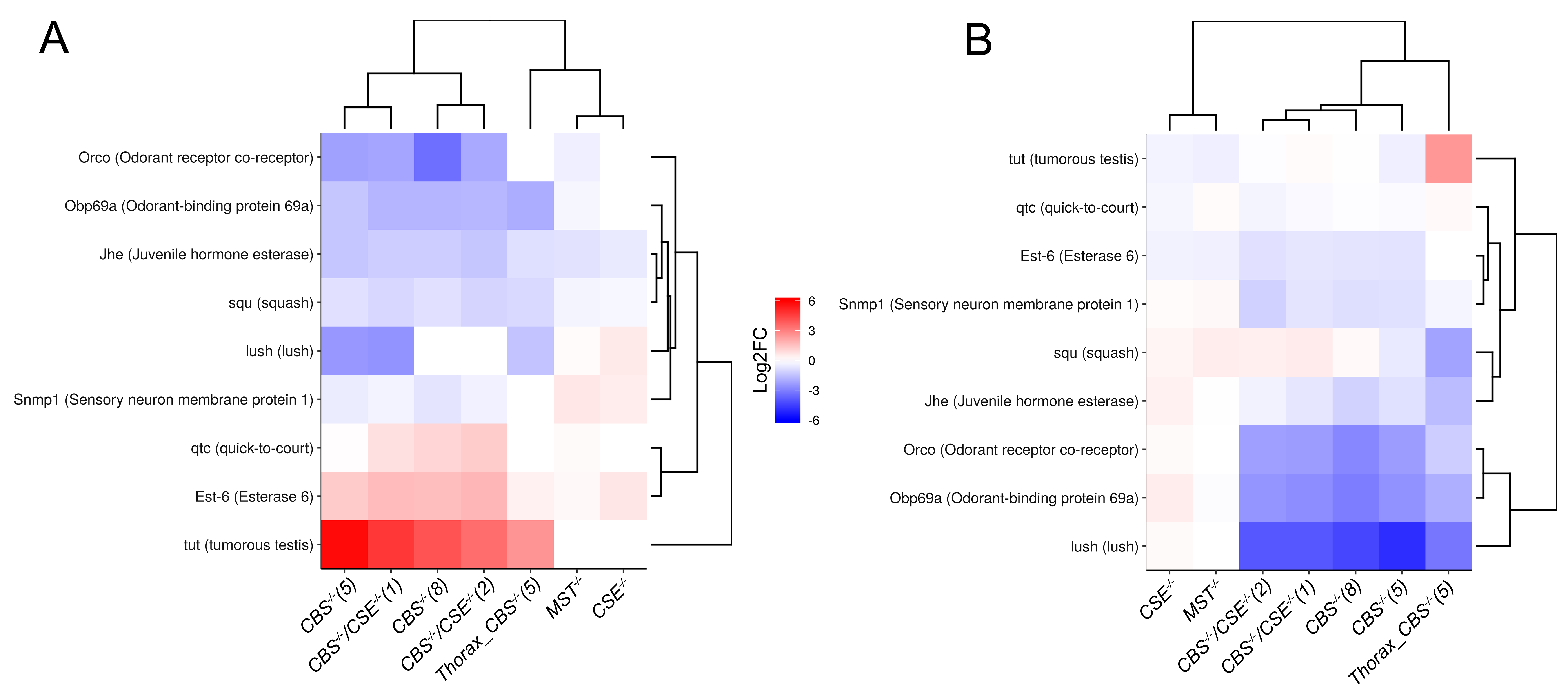

Supplement: Multimedia component 1 [file mmc1.zip › Figure_S10.tif]

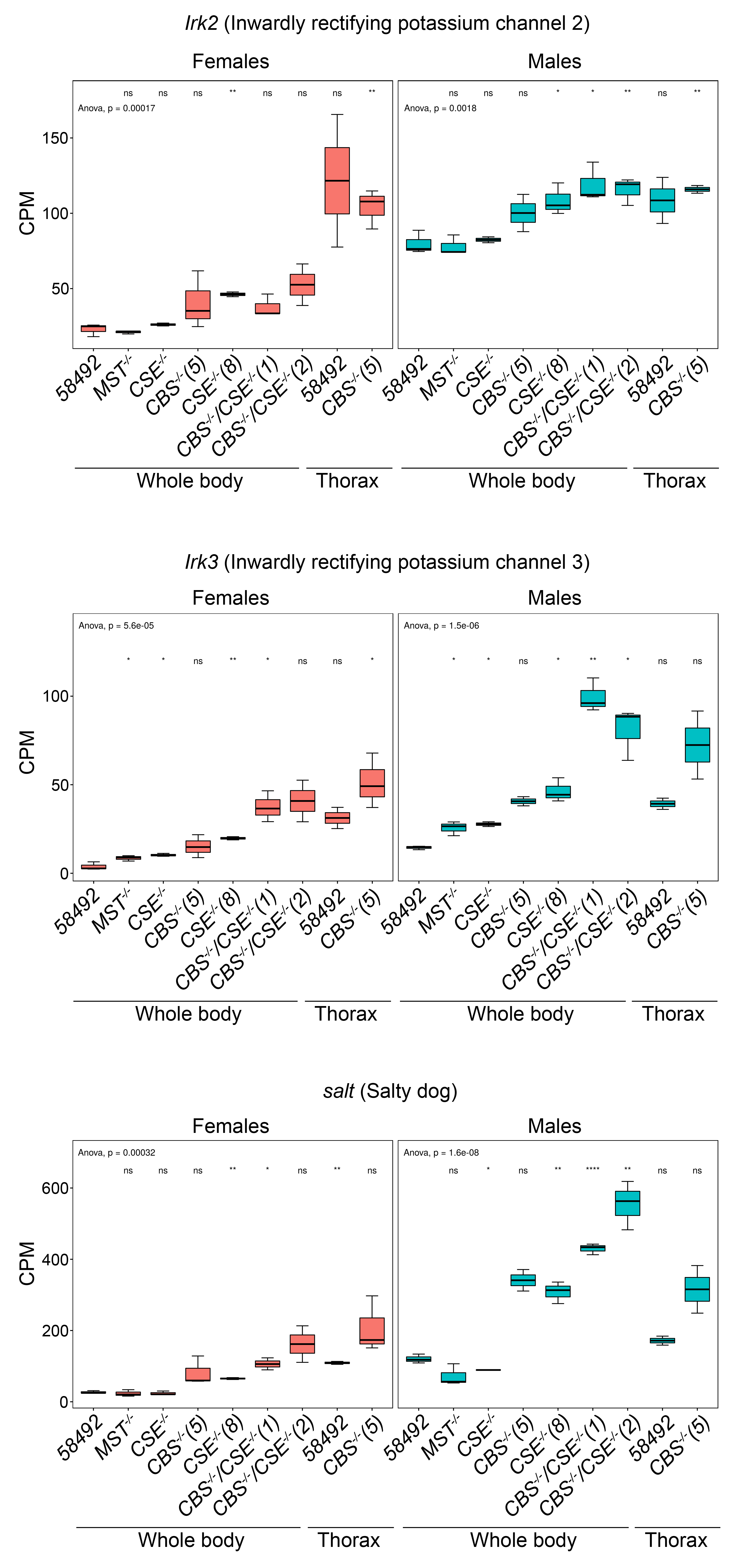

Supplement: Multimedia component 1 [file mmc1.zip › Figure_S11.tif]
